# Diffusion of Carbamazepine in Hydrophobic Zeolites: A Comparative Study Using Classical and Machine‐Learned Potentials

**DOI:** 10.1002/chem.71048

**Published:** 2026-04-24

**Authors:** Jakob Brauer, Richard Kendra, Carlos Bornes, Lukáš Grajciar, Michael Fischer

**Affiliations:** ^1^ Crystalline Microporous Materials Crystallography and Geomaterials Research Faculty of Geosciences University of Bremen Bremen Germany; ^2^ Bremen Center For Computational Materials Science and MAPEX Center for Materials and Processes University of Bremen Bremen Germany; ^3^ Department of Physical and Macromolecular Chemistry Charles University Praha Czech Republic

## Abstract

Hydrophobic zeolites are promising adsorbents for the persistent pollutant carbamazepine (CBZ), yet diffusion within their confining pores remains poorly understood. Computational studies often rely on static interaction energies, conveying only a static picture. We employ umbrella sampling simulations to obtain free energy surfaces (FES) of CBZ diffusion in a range of all‐silica zeolites. Results from a classical force field description are compared with a fine‐tuned neural network potential (MACE). While both methods show qualitative agreement, the MACE potential mostly predicts higher activation barriers, which we attribute to the more accurate representation of the energy penalty of close atomic contacts at the transition states. MACE simulations show that CBZ can become kinetically trapped in higher‐energy, metastable orientations after a transition state. We propose that exergonic adsorption from an aqueous phase would populate an ensemble of lowest‐energy and metastable states, providing a plausible kinetic pathway for diffusion, with a lowering of the effective activation barrier with respect to higher‐energy states. The diffusion of CBZ is governed by a complex landscape of translational and rotational barriers, a picture only accessible by going beyond an interaction energy‐based description. This work supports the rational choice of shape‐selective zeolites as effective adsorbents for environmental remediation.

## Graphical abstract

Can hydrophobic, shape‐selective zeolites efficiently remove the persistent pharmaceutical CBZ from water? This work moves beyond the static picture of interaction energies by modeling diffusion with umbrella sampling and machine‐learned potentials. Even high intrinsic diffusion barriers can be overcome through exergonic adsorption from water, yielding insight for the design of efficient adsorbents for wastewater treatment.

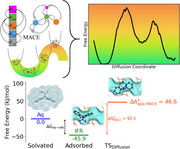

## 1 Introduction

Carbamazepine (CBZ, 5*H*‐dibenzo[*b*,*f*]azepine‐5‐carboxamide, C_15_H_12_N_2_O) is an anticonvulsant and anti‐epileptic drug which is mainly used in the preventive treatment of seizures but also prescribed for the treatment of schizophrenia, depression and pain syndromes due to its psychotropic effects [1, 2]. Due to its broad applicability, about 3,000,000 prescriptions for the drug are issued annually in the US alone [3]. Global consumption is steadily increasing with over 1000 tons being globally consumed in 2015 and a yearly increase in consumption of roughly 100 tons [4]. Resulting from its intensive utilization and incomplete metabolization, CBZ dissipates into the environment, mainly through municipal and hospital wastewaters, and is highly persistent and resistant to conventional wastewater treatment processes, so much that it has been adopted as a molecular anthropogenic marker [5, 6, 7, 8]. The concentrations that occur in surface waters and drinking waters are not considered harmful to humans, however effects onto aquatic species have been observed, for example, by impacting the development of fish embryos and the behavior of larvae, which can induce dramatic cascade effects by disturbing the balance of local ecosystems [9, 10, 11, 12]. In addition to the persistence of CBZ, its metabolites upon microbial breakdown and its photolytic transformation products have been shown to be equally or even more hazardous toward aqueous environments, rendering degradation‐based water treatment unfit to resolve the issue [6, 10, 13].

**FIGURE 1:**
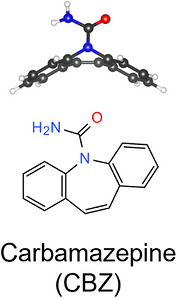
CBZ in stick‐and‐ball representation (black: C, white: H, blue: N, red: O) and Lewis structure.

Adsorption‐based water treatment is a promising approach to remove even trace amounts of CBZ and also its metabolites from water. Activated carbon materials, though being the state‐of‐the‐art solution, suffer from unselective adsorption and passivation by natural organic matter in complex water matrices [14]. Hydrophobic zeolites offer high selectivities and similar or even higher adsorption capacities than activated carbons [15, 16]. Because of their higher synthetic cost zeolites do not constitute a replacement of activated carbon at the moment, but their selectivity and regenerability could make them relevant for targeted applications [17]. Their crystalline structure and defined pore topologies are already exploited and widely used in catalytic and separation applications [18, 19]. Experimental studies have investigated the adsorption of CBZ in hydrophobic zeolites, such as silicalite‐1 (MFI topology), mordenite (MOR topology) or zeolite Y (FAU topology) and have demonstrated for zeolite Y that the adsorption efficiency in pure water and real wastewater samples is largely unaffected [20]. Other works have investigated the adsorption onto transition metal cation‐exchanged or surfactant cation modified zeolite Y or modified zeolitic tuff with surfactants and reported an enhanced adsorption performance by incorporating surfactants [21, 22]. Complementing the experimental studies, CBZ has been featured in computational screening approaches and in‐depth studies that investigated the static interactions of CBZ with all‐silica zeolites on the classical forcefield level of theory [23, 24] and with density functional theory (DFT) [25]. A key finding across these studies is the exceptionally strong shape‐topology relation, showing the interaction energy is maximized when there is a match between the relatively rigid angled geometry of CBZ (see Figure 1) and the zeolite pore topology.

These experimental and computational studies primarily focus on the maximum adsorption capacity at thermodynamic equilibrium or the interactions in equilibrium configurations. In practical applications, such as continuous‐flow water treatment columns, the process is kinetically limited and rarely reaches equilibrium. Therefore, the overall efficiency is determined not just by how much can be adsorbed, but by how fast it can be adsorbed. This rate is ultimately governed by the intracrystalline diffusion of CBZ through the zeolite pore network. Understanding the diffusion pathways and their associated free energy barriers is thus essential for evaluating and designing effective zeolite adsorbents.

Experimental study of diffusion for nongaseous molecules can be challenging. Two of the most prominent methods for this are pulsed field nuclear magnetic resonance (NMR) spectroscopy [26, 27], which can suffer from short spin‐relaxation times and internal magnetic field artifacts at the interfaces of the system, and quasi‐elastic neutron scattering [28], which requires complex models to resolve the translational motion from rotational and vibrational contributions. Exemplary studies have been reported utilizing fluorescent probe molecules in conjunction with interference microscopy and infrared micro‐imaging to investigate and visualize diffusion in zeolite Beta (BEA topology) [29]. Classical force field simulations offer an intuitive and in principle easily accessible way to study diffusion in zeolites. Since the time‐evolution of a system is explicitly modelled, the movement of the molecule over time can be directly connected to macroscopic diffusion coefficient. Studies focusing on the diffusion of simple and rather small organic molecules like benzene [30], xylenes [31], methane, and mercaptan [32] in zeolites have successfully employed the Einstein relation, which connects the mean‐squared displacement (MSD) of a molecule over the course of a simulation with a self‐diffusion coefficient [33]. For this to be valid, a roughly linear development of the MSD over time has to be given.

With the dimensions of the molecule approaching the pore diameter, the barriers associated with diffusion can become high (>100 kJ/mol) [34]. Depending on the energetic barrier of diffusion, accessing such an event by equilibrium molecular dynamics (MD) can be rare or even impossible. To enable statistically significant sampling of these rare‐events, an enhanced sampling strategy needs to be adopted, such as the popular umbrella sampling [35]. In these enhanced sampling simulations, the rare diffusion event can be intensively studied by biasing the molecule to sample configurations along the diffusion coordinate, the free energy barrier can be accessed and conclusions for the real‐world diffusion can be inferred. Umbrella sampling has been widely applied to investigate and understand adsorption and diffusion processes in many systems, ranging from the investigation of anion transport through membrane proteins and bio‐membranes [36, 37] to the modelling of the adsorption and diffusion of perfluorooctanoic acid from water into the pores of zeolite Beta [38].

While most works that have employed umbrella sampling relied on classical interatomic force fields to be able to access timescales that ensure a sufficient sampling of the phase space, recently the neural network potentials (NNPs) have gained popularity [39]. NNPs are typically trained to reproduce *ab initio* energies and forces, outperforming classical forcefields by intrinsically including polarizability, reactivity and a proper representation of complex chemical environments. Another crucial advantage is the evasion of stiff analytical expressions for chemical bonds, angles, dispersion interactions, etc., which are known to perform well in the equilibrium region but often fail in higher‐energy regions of the potential energy surface [40]. Among the manifold of NNP architectures, the recently reported MACE potentials stand out due to their transferability between chemical systems and the possibility to finetune foundation models that are trained on large databases [41, 42].

In this work we utilize umbrella sampling simulations employing both classical force field parameters and a finetuned MACE potential to obtain kinetic information about the diffusion process of CBZ in hydrophobic zeolites. This insight provides a thorough understanding of these systems and aims to ultimately inspire the development of selective water treatment applications to remove CBZ from water resources.

## 2 Computational Methods and Details

The starting configurations of CBZ in the eight considered all‐silica zeolites (JZT, CFI, FAU, IFR, MOR, BEA, MWW, MFI topologies) were taken from our previous work [24]. The MD simulations at the classical force field level of theory were carried out using the LAMMPS software package [43] in the 29Aug2024 version. In these simulations, the parameters to describe the guest molecule were taken from the OpenFF Sage 2.0 force field [44] and the parameters describing the bonds, angles, Lennard‐Jones (LJ) parameters, and partial charges of the zeolite frameworks were adopted from Emami et al. [45]. This combination of parameters was validated against DFT interaction energies (at rev‐vdW‐DF2 functional level) in our previous work [24] and also showed good agreement with the free energies derived from adsorption isotherms, which we reported recently [46]. Also, since we employ classical FF simulations only to identify systems for further study with the machine‐learned potential, no systematic comparison among different sets of classical FF parameters is performed.

All classical force field MD simulations were performed in the *NVT* ensemble, using a Nose‐Hoover thermostat with a damping factor of 100 timesteps at 300 K [47, 48]. Dispersion was modelled using a 12‐6 LJ potential with a cut‐off of 12 Å, shifted to zero at the cut‐off and employing Lorentz‐Berthelot mixing rules. Electrostatics were treated explicitly up to a cut‐off of 12 Å and after that with the particle‐particle‐particle‐mesh method [49] with a relative cutoff of 10^−6^.

Several kinds of classical force field MD simulations were performed. Free energy perturbation (FEP) simulations were carried out to obtain adsorption free energies with respect to the vacuum state, which were done in line with the setup used in our previous work [24]. To investigate the mobility of CBZ in a subset of five zeolites (JZT, CFI, FAU, IFR, BEA), equilibrium classical MD were performed with a timestep of 4.0 femtoseconds, employing the SHAKE algorithm [50] to constrain all bonds involving hydrogen atoms and the H‐N‐H angle of the amide group. We would like to point out that these simulations served only as a qualitative measure of the mobility of CBZ in the pores and are not evaluated further. Umbrella sampling simulations on the classical force field level of theory were done with a timestep of 0.5 fs.

MD simulations employing a MACE [41] potential were carried out within the Atomistic Simulations Environment (ASE) [51] in the *NVT* ensemble, using a Langevin thermostat with a friction coefficient of 0.01 at 300 K with a timestep of 0.5 fs. The MACE potential employed in the simulations is a finetuned foundation model (mace_mp_0b2) [42]. The data used for the finetuning and the validation of the finetuned model is described in section S1 of the supporting information.

Harmonic restraints were applied to the classical force field and to the MACE MD simulations with the PLUMED library in the version 2.8.4 [52]. A harmonic umbrella potential with the corresponding force constant [53] acting on the center of mass (COM) of the CBZ molecule was used, which was moved from the initial position of the COM to the respective position of the umbrella window over the duration of 1 ps. Subsequently, each simulation was equilibrated for 4 ps, after which the geometry was optimized while keeping the restraining potential in place, followed by a production run for 100 ps for each window. Additional equilibration and production durations were considered to ensure the validity of the chosen setup, a comparison is included in the SI section S2.

Simulations of diffusion in the IFR framework using the MACE potential showed significant kinetic trapping, where the guest molecule would remain in high‐energy, metastable orientations even after the standard equilibration protocol. This resulted in poorly converged free energy surfaces (FES) and unphysically high energy differences between equivalent local environments. To overcome this severe sampling issue, a simulated annealing protocol was employed during the equilibration phase for this system. After the initial positioning and optimization, each window was equilibrated for 7.5 ps at an elevated temperature of 600 K at the restrained position. This allowed the system to rapidly overcome local rotational barriers and explore the conformational space. Subsequently, the particle velocities in the system were re‐initialized from a Maxwell‐Boltzmann distribution at 300 K. The production data for the FES calculation was collected exclusively at 300 K. This procedure ensures that the resulting FES is a valid representation of the canonical ensemble at the target temperature, with the annealing step serving only as a more efficient method to achieve proper equilibration in a rugged energy landscape.

Umbrella sampling data were decorrelated and analyzed using the timeseries module and the multistate Bennet‐Acceptance‐Ratio (MBAR) method in pymbar python package [54, 55, 56]. Force constants and window spacing were iteratively optimized using the Optimal Grid Refinement (OGRe) algorithm [57]. The accuracy of the FES was assessed using an automated in‐house implementation of the OGRe protocol, which evaluates: (i) overlap between neighboring windows, (ii) confinement within window boundaries, and (iii) consistency with the reweighted distribution. In OGRe, each iteration applies these metrics to refine the setup: insufficient overlap triggers insertion of a new window at the midpoint between neighbors; poor confinement increases the corresponding force constant; and low consistency likewise increases the force constant. This adaptive scheme was reimplemented according to the original paper by Borgmans et al. [57]., but we employed the MBAR method for postprocessing instead of weighted histogram analysis method (WHAM) [58]. Complete OGRe metrics and reproduction scripts are provided in the Supporting Information. Initial force constants in umbrella windows varied from 100 kJ/(mol∙Å) in extra‐large pore zeolites to avoid artifacts from flat potential energy surface up to 3000 kJ/(mol∙Å) to model the diffusion through the (in principle too) narrow 10 MR channels of MWW and MFI. The activation free energy $\Delta A_{A\to B}^{‡}$ was determined by accounting for the full partition function of the reactant basin as well as of the curvilinear nature of the collective variable [59], rather than just taking the difference of the minimum and the maximum of the FES, by Equations (1 and 2).

(1) $$\Delta A_{A\to B}^{‡}=-k_{B}T\ln\frac{\rho \left(\xi^{‡}\right)\lambda_{\xi }}{P_{A}}$$

(2) $$\lambda_{\xi }=\frac{h}{\sqrt{2\pi m_{\xi }k_{B}T}}$$

Here, $k_{B}$ corresponds to the Boltzmann constant, T is the temperature, $\xi^{‡}$ is the position of the transition state along the collective variable and $\rho \;\left(\xi^{‡}\right)=\exp\frac{-A(\xi^{‡})}{k_{B}T}$ is the unnormalized probability density at the transition state. $P_{A}$ is the total integrated probability of being in the reactant state A, and $\lambda_{\xi }$ corresponds to the thermal de‐Broglie wavelength associated with the effective mass $m_{\xi }$ of the collective variable at the transition state.

## 3 Results and Discussion

### 3.1 Adsorption Energy and Mean‐Squared Displacement of CBZ in Zeolites

**FIGURE 2:**
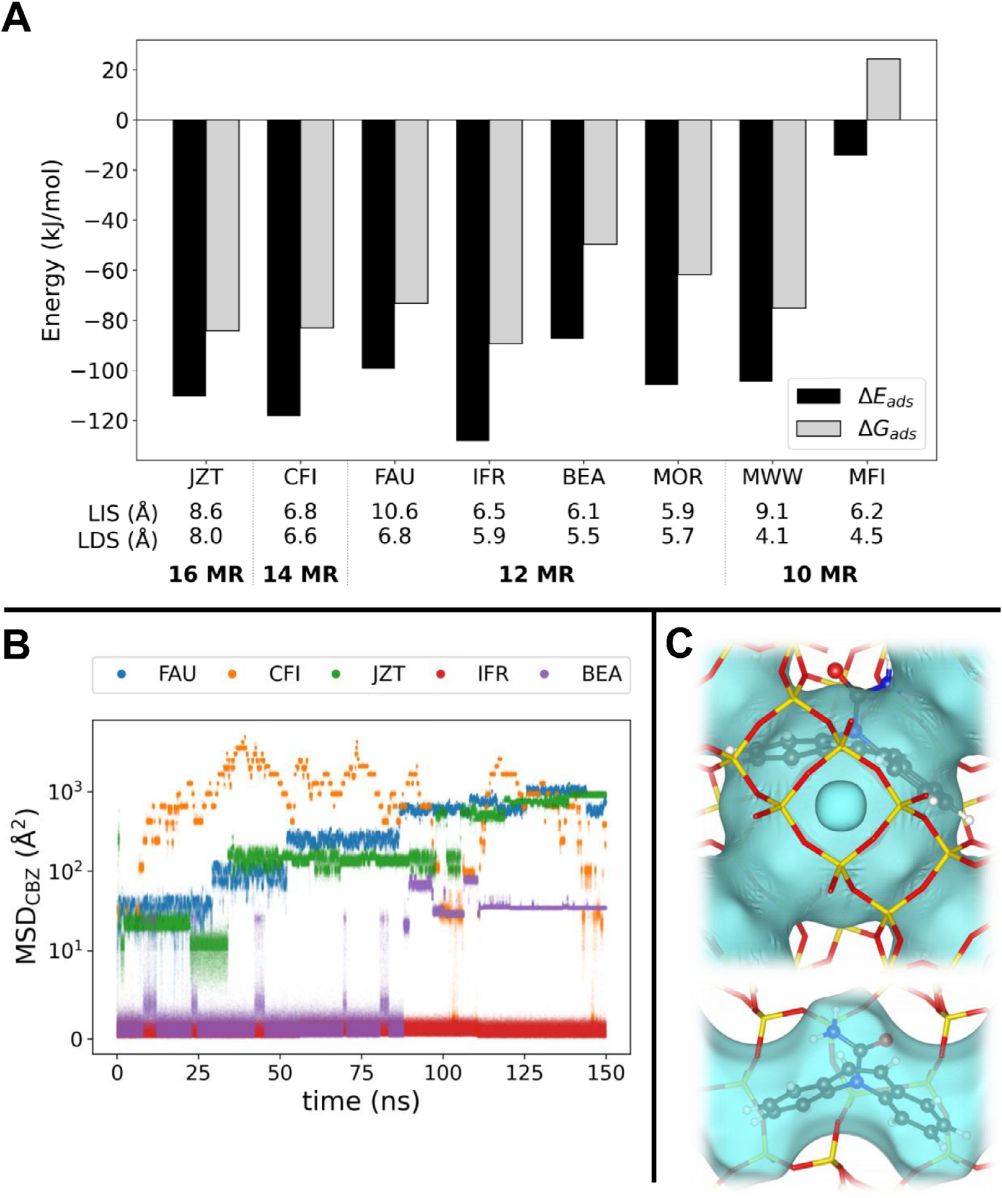
(A): Bar plot showing the Boltzmann weighted adsorption energies from optimized adsorption configurations [24] and the adsorption free energy from FEP simulations. Along with the framework type code (FTC), the largest included sphere diameter (LIS) and largest diffusing sphere diameter (LDS) (calculated with the zeo++ package [60]) of the respective zeolite framework is shown as well as the classification into *N*‐MR pores. (B): Mean squared displacement of the CBZ molecule over the course of a 150 ns MD trajectory at 300 K in the respective zeolite frameworks. (C): Adsorption configurations of CBZ in FAU and IFR. The light blue pore outlines correspond to the electron density contours calculated for the zeolite frameworks, plotted with an isovalue of 0.0002 e/Å^3^.

As discussed in the introduction, the interaction of CBZ with a zeolite pore is strongly dependent on the match between the molecular shape and the pore topology. This is demonstrated by considering the classical FF Boltzmann‐weighted interaction energies (taken from our previous work [24]) and the adsorption free energies of CBZ in 8 different all‐silica zeolite frameworks, diverse in their pore size and pore shape. The frameworks and their respective pore sized denoted as *N* membered‐rings (MR) included in this study are: JZT (16 MR), CFI (14 MR), FAU, IFR, BEA, MOR (12 MR), and MWW, MFI (10 MR). The Boltzmann‐weighted interaction energies and the adsorption free energies are shown in Figure 2A, which includes the largest included sphere diameter (LIS) and the largest diffusing sphere diameter (LDS / pore limiting diameter).

As the adsorption free energy accounts for the loss in entropy upon confinement, the adsorption free energy is consistently smaller than the interaction energies and decreases more strongly in strongly confining small pores. The most strongly interacting framework is IFR, because the sinusoidal channels of IFR allow for a tight fit of the CBZ molecule. In contrast, the adsorption free energy in the MOR and BEA framework are the among the weakest, due to the straight channels of these zeolites, that force the molecule to flatten, leading to a less favorable interaction. In the narrow 10 MR pores of MFI the insertion of the molecule is only possible at an intersection between the straight and the sinusoidal channels and only under significant deformation of the molecule, leading to a positive adsorption free energy. The strong interaction with the MWW framework can be attributed to the large channel intersections, indicated by the LIS of 9.1 Å for this framework, however diffusion through the 10 MR channels is likely not possible as the LDS of 4.1 Å is smaller than the diameter of the CBZ molecule.

For a subset of zeolite frameworks, equilibrium MD simulations were employed to probe the mobility of the molecule in different pore systems. The MSD (mean‐squared‐displacement) of the center‐of‐mass (COM) of CBZ (with respect to its initial position) over the course of a 150 ns trajectory at 300 K is shown in Figure 2B. For FAU and JZT (blue and green) which both have a 3D connected pore space, the MSD shows a stepwise evolution. For FAU this corresponds to occasional jumps between supercages through cage‐connecting 12 MR windows. Similarly, the 16 MR channels of JZT form large intersections, between which the molecule transitions. CFI (orange) consists of 1D straight 14 MR channels, which is reflected in the increase and decrease of the MSD. The molecule can only move either in one or the other direction, thus it moves away and back to its initial position. For IFR (red) the MSD stays very close to 0, indicating that the molecule hardly moves at 300 K. In zeolite Beta (BEA, purple) the molecule occasionally moves away from its initial position, but the jumps are very localized and quite rare. The adsorption configurations and available pore space for the adsorption in FAU and IFR are shown in Figure 2C. For a more intuitive measure of the mobility, we estimate the absolute displacement from the maximum value of the MSD(*t*) over each whole trajectory by assuming a Gaussian distribution of the displacement of CBZ. This value reflects the maximum distance that the CBZ molecule traveled from the origin during the simulation. The square‐root of the MSD is corrected by the pre‐factor $\frac{4}{\sqrt{6\pi }}$ for 3D motion in the interconnected pores of FAU, JZT and BEA, in the channels of CFI and IFR 1D motion is assumed, since the pores are not interconnected in these frameworks, giving a correction factor of $\sqrt{\frac{2}{\pi }}$. The reasoning for the pre‐factors is given in the SI, section S3. For each of the systems the estimated absolute displacements are: 34.5 Å (FAU, MSD_max_ = 1405.1 Å^2^), 57.2 Å (CFI, MSD_max_ = 5136.7 Å^2^), 33.5 Å (JZT, MSD_max_ = 1322.2 Å^2^), 2.0 Å (IFR, MSD_max_ = 6.2 Å^2^), 10.2 Å (BEA, MSD_max_ = 123.2 Å^2^).

The analysis of the MSD over time indicates that the mobility of CBZ is significantly hindered in zeolites with narrower pores apertures on the nanosecond timescale that can be accessed with these equilibrium simulations. In the wider pores, some diffusion is observed, but for an evaluation using the Einstein relation, a roughly linear MSD over time would be necessary, which is not the case even for the frameworks with the largest pores. A direct relation between adsorption energy and mobility is not observed, for example, IFR and CFI exhibit the strongest interactions with CBZ, yet show vastly different molecular mobilities (see Figure 2B). This demonstrates the necessity to explicitly evaluate diffusion barriers to assess the mobility of CBZ.

### 3.2 Classical FF Umbrella Sampling Simulations of CBZ in Zeolites

To investigate the diffusion of CBZ in these zeolites, we employ umbrella sampling simulations to obtain free energy profiles along the diffusion coordinate (see below). By applying transition state theory, the activation barriers can be translated to self‐diffusion coefficients (Equations 3 and 4), but already from the height of the barrier an estimate can be made if the process is likely to happen at ambient temperature. For zeolites with one‐dimensional channels, the collective variable is defined as the position of the molecule's center of mass projected onto a vector along the channel axis. For FAU the diffusion is modelled as a jump from the center of one supercage to the next through a 12 MR window connecting the supercages. For JZT the low‐energy adsorption site for CBZ is inside a 16 MR ring, so the diffusion path is defined as the vector from the center of one 16 MR to the next one, passing through the wide channel intersection of the intersecting 16 MR channels.

**FIGURE 3:**
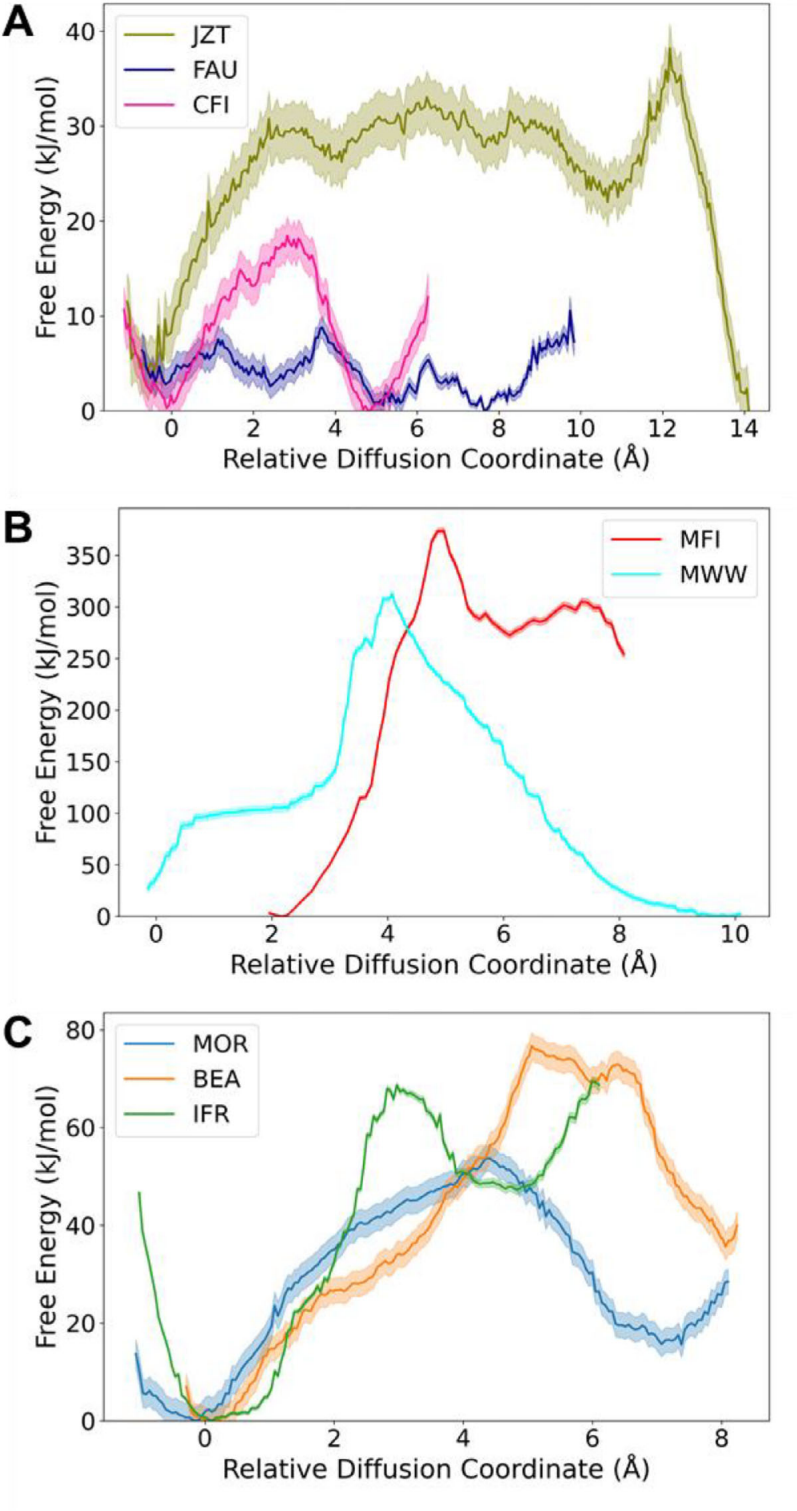
(A) FES of CBZ diffusion through JZT (olive), FAU (brown) and CFI (magenta) (B) FES of CBZ diffusion through MFI (red) and MWW (cyan). The low barriers in plot A are in line with the MSDs in Figure 2. The high barriers in plot B agree with the experimental observation that CBZ does not get adsorbed into 10 MR pores [20]. (C) FES of CBZ diffusion through IFR (blue), MOR (orange), and BEA (green). All shown FES are obtained from classical FF umbrella sampling simulations.

First the frameworks with pore‐limiting diameters of more than 6 Å are considered (Figure 3A). Judging from the MSD of CBZ in CFI, FAU, and JZT (Figure 2B) a relatively small diffusion barrier is expected for these systems. In FAU (Figure 3A, dark blue), the FES is largely flat with the highest barrier of ∼10 kJ/mol at around 4 Å arising from the passage through the supercage connecting 12 MR window. We note that an activation barrier of ∼10 kJ/mol theoretically corresponds to picosecond‐scale crossing rates, which contrasts with the nanosecond‐scale jumps observed in the unbiased MSD (Figure 2B). This discrepancy arises because the 1D collective variable only biases the translational motion of the molecule but might insufficiently sample the random rotational dynamics of CBZ exploring the supercage. In the unbiased system, the bulky CBZ molecule spends considerable time sampling the supercage before randomly achieving the precise orientation required to pass through the 12 MR window, which ultimately governs the evolution of the MSD. The low activation barrier is in line with experimental observations reported by Martucci et al. stating that the adsorption of CBZ with FAU from water was equilibrated after 2 h of contact time [20].

The clear periodicity of the FES in CFI (Figure 3A, pink), can be explained from the pore wall architecture. The walls of the 14 MR channels consist of alternating, edge‐sharing 6 MRs, which form “bumps” in the pore wall for the amide group of CBZ to point into, offering distinct energetically favorable adsorption sites, and slightly more narrow parts of the channel as barriers. The difference between LIS and LDS in the CFI framework is merely 0.2 Å, which is reflected in the small activation barrier of about 20 kJ/mol.

Considering JZT (Figure 3A, olive), a steep rise of the FES occurs right after the stable adsorption site in the 16 MR. This energy barrier corresponds to the desorption from the stable adsorption site into the vast channel intersection, where CBZ is surrounded by fewer framework atoms, resulting in weaker dispersion interactions and thus a restoring force toward the initial adsorption site. After desorption from the adsorption site, the FES is relatively flat until the next 16 MR is reached after ∼14 Å, which leads to the energy decreasing toward the initial state. In contrast to the diffusion through FAU and CFI, where passing through the narrower part of the pore is the energetically demanding process, in JZT the desorption from the adsorption site poses the most significant energetic barrier. This finding underscores the limitations of relying solely on static geometric descriptors like LIS and LDS and static data like adsorption energies. Only an explicit simulation of the free energy landscape can reveal the distinction that for JZT, the rate‐limiting step is desorption from the stable site rather than passage through the narrowest pore aperture. For those three frameworks, an effectively barrierless diffusion at room temperature can be expected.

Diffusion in the 10 MR zeolites is depicted in Figure 3B. With an LDS of 4.1/4.5 Å, the simulations predict an activation barrier of more than 300 kJ/mol for CBZ to pass through the narrowest part of the pore, which can be considered impossible at room temperature. This computational result is in line with experimental observations, where the very low uptakes of CBZ by MFI‐type zeolites have been attributed to adsorption on the external surface only, with the pores considered too narrow for CBZ to enter [20].

Next, the zeolite frameworks with LDS of around 5 to 6 Å (IFR, MOR, and BEA) were considered. For BEA and IFR the MSD of CBZ over the 150 ns MD trajectory showed only negligible mobility. The adsorption free energy of CBZ in these zeolites varies widely, with IFR having the value with –89.3 kJ/mol, while BEA and MOR show a weaker adsorption free energy of –49.7 kJ/mol and –61.6 kJ/mol, respectively. The FES along the diffusion coordinate are shown in Figure 3C.

All FES of the diffusion through the 12 MR zeolites do not end up in the same energetic minimum the jump‐diffusion started from. The reasons for this are discussed in‐depth in the section S4 of the supporting information. Since the diffusion through the rather tight 12 MR pores involves strong deformations, given the activation barriers of more than 50 kJ/mol, it is debatable if the chosen combination of force field parameters can accurately reflect the energetics of these strained configurations, especially for a complex molecule like CBZ [40].

Thus, we neglect the actual activation energies from the classical FF simulations and just utilize them to identify distinct jump‐like diffusion behavior, that is likely possible under ambient conditions. The zeolite frameworks CFI, BEA, MOR and IFR are further investigated with umbrella sampling simulations employing a finetuned MACE potential. Still, we would like to highlight that on the classical FF level of theory, diffusion seems to hardly be affected by the differences in interaction energy. It is especially worth highlighting, that despite the large difference in adsorption energy between IFR (strongest interaction) and BEA (weakest interaction within 12 MR zeolites) (see Figure 2C) the energetic barrier is similar.

### 3.3 Investigating Thermally Accessible Jump‐Like Diffusion With a Finetuned MACE Model

**FIGURE 4:**
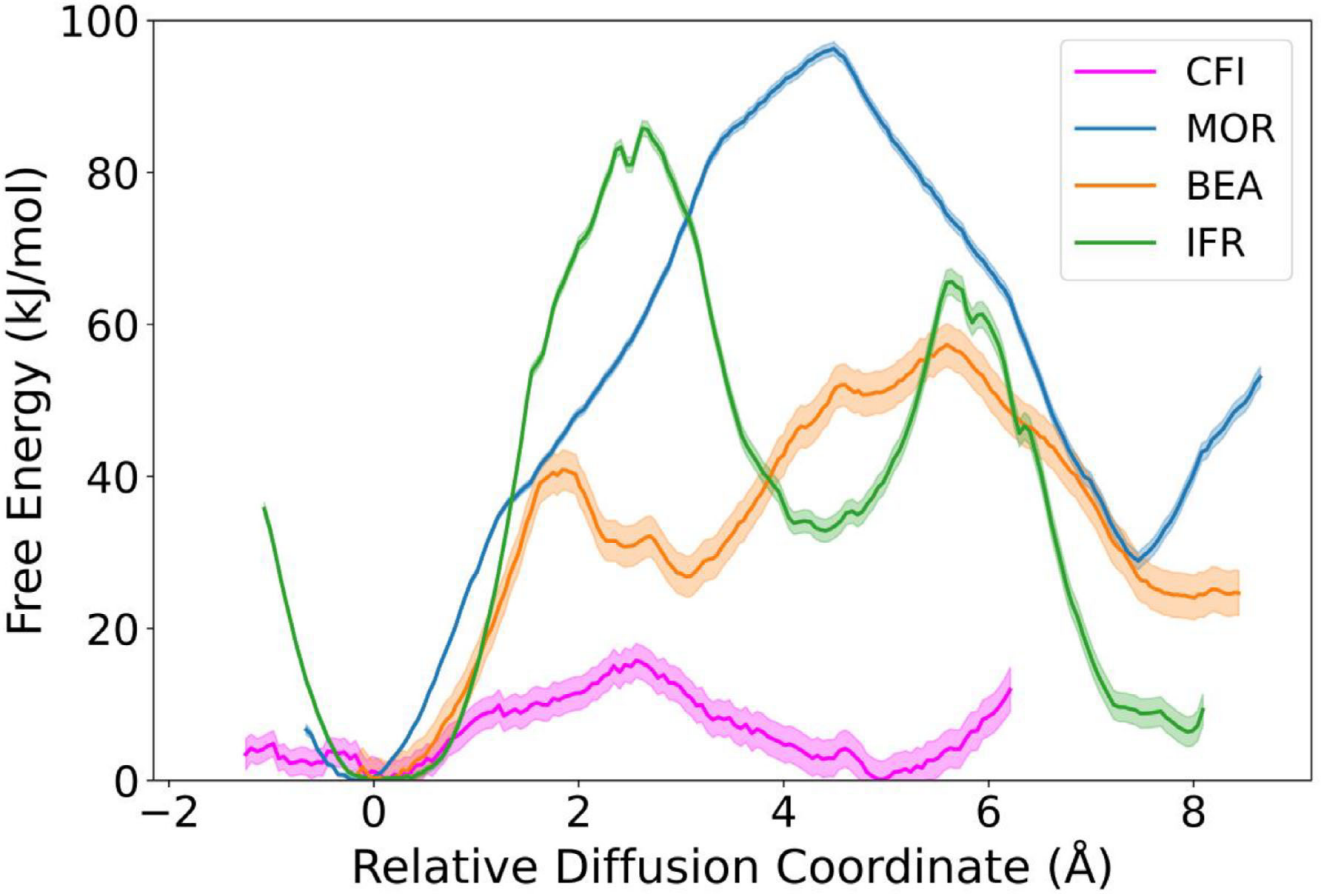
FES of CBZ diffusing through CFI (magenta), MOR (blue), BEA (orange), and IFR (green) obtained from MACE simulations.

The FES presented in Figure 4 are obtained with varying equilibration strategies and production times. Since they depend on the specifics of the system, these variations were necessary to obtain a better description of the respective diffusion jump. The FES depicted in Figure 4 will only shortly be discussed here, an in‐depth discussion on the difference among the equilibration strategies and production times as well as a detailed comparison of the FES from classical FF and MACE simulations is provided in section S5 of the supporting information. The comparison of the two levels of theory shows that the two methods agree very well in largely unconstrained environments, while the results differ in strongly confining pores.

The FES for the diffusion through CFI and BEA were obtained with the standard simulation strategy of 5 ps equilibration followed by 100 ps of data production. The depicted FES for the diffusion through MOR was obtained with a production duration of 200 ps. For the diffusion through IFR, CBZ became trapped in high‐energy metastable states after the transition state with the standard simulations protocol. To overcome this, an annealing step was introduced after the CBZ molecule was moved to the position of the umbrella window and prior to the data production and data was produced for 200 ps. For a detailed discussion of the effects of this strategy, see section S5 of the supporting information.

For the diffusion in CFI (Figure 4, magenta) an activation free energy of about 21 kJ/mol is obtained with the MACE potential, which is almost identical to the classical FF result. Also, the FES ends up in the same energetic minimum after the diffusion jump, once more indicating that describing diffusion in a less confined environment, such as the wide 14 MR channels of CFI, is relatively straightforward. From the low activation free energy, we infer a largely instantaneous diffusion.

In the narrower 12 MR channel of BEA, IFR, and MOR, the diffusion barriers are higher and the FES show some asymmetry, similar to the classical FF FES. This asymmetry is further discussed in the Section S5. In the case of BEA (similar to the classical FF simulations) it is attributed to a 90° rotation of the whole CBZ molecule, at the commensurable adsorption site compared to the initial configuration (see Figure 4 (orange) and Figure S7B).

In MOR (Figure 4, blue) the energy at the commensurable adsorption site is about 30 kJ/mol higher than in the initial configuration. From a data production duration of 100 to 200 ps this difference decreases (Figure S7C), indicating that high‐energy configurations after the diffusion jump strongly influence the height of the second minimum and that a longer simulation would end up in the same minimum as the initial configuration. However, the height of the diffusion barrier is largely independent of the increased production duration (varying only by about 1 kJ/mol), which shows that the transition state is sufficiently sampled within the simulation and the activation free energy from the initial adsorption site to the highest point of the barrier can be considered as an upper bound for the activation free energy of a diffusion event.

The periodicity of the FES for the IFR diffusion is in line with the geometry of the sinusoidal channels of IFR with alternating protrusions on the opposite sides of the channel (Figure 4, green). CBZ rotates along the diffusion path by 180° to fit into the next protrusion and rotates again back into its initial position in the second minimum of the IFR FES (see Figure S7D). It was necessary to include an annealing step prior to the data production to allow the CBZ molecule to rotate perpendicular to the diffusion coordinate within the equilibration time.

From the asymmetric FES in the 12 MR zeolite cases, it is apparent that describing diffusion in a more confined environment comes with significant additional challenges that can require an adaptation of the simulation strategy such as increased data production time or high‐temperature equilibration. While in theory the reaction free energy of a jump‐diffusion from one adsorption site to a commensurable site should be zero, the slow equilibration is not necessarily only a computational artifact, but can also be a reflection of the behavior of such systems. In strongly confined systems a diffusion jump induces deformations in the molecule and the zeolite framework, which can be persistent and require significant timespans to relax. This additional potential energy in the system can lead to the next diffusion jump being energetically more favorable than the initial jump, effectively lowering the mean diffusion barrier and increasing the self‐diffusion coefficient. In the next section the activation free energies for the forward jump (A→B) and the backward jump (B→A) as well as the average activation free energy (A↔B) are converted to self‐diffusion coefficients and half‐lives.

### 3.4 Deducing Diffusion Properties From MACE Activation Free Energies

The resulting activation free energies ($\Delta A_{A\to B}^{‡}$) from the MACE simulations, the calculated self‐diffusion coefficients (D) and half‐lives ($t_{1/2}$) are summarized in Table 1. Self‐diffusion coefficients (Equation 4) are calculated from rate‐constants (k) obtained from the Eyring equation (Equation 3), taking the transmission coefficient κ as 1 and multiplying the rate constant by the squared length of the jump between the minima of the FES (*l*) times ½ since the diffusion through the channels implies a 1D diffusion [61]. It has to be noted that despite modelling the diffusion as 1D, the BEA framework consists of interconnected 12 MR channels, which would enable 2D or 3D diffusion. By assuming a first‐order behavior, where the intra‐crystalline diffusion is the rate‐limiting step, we can also obtain the half‐life (*t_1/2_*) of the adsorption from the rate constant (Equation 5).

(3) $$k=\kappa \frac{k_{B}T}{h}\exp\left(-\frac{\Delta A_{A\to B}^{‡}}{RT}\right)$$

(4) $$D=\frac{1}{2}kl^{2}$$

(5) $$t_{1/2}=\frac{\mathrm{In}\left(2\right)}{k}$$

**TABLE 1:** Activation energies (*∆A^‡^*) along the CV in positive and negative direction obtained from MACE simulations for the frameworks CFI, BEA, MOR and IFR. Based on *∆A^‡^* self‐diffusion coefficients (*D*) are calculated from the Eyring equation (with a transmission coefficient of 1) for the forward and backward jump and for the average of both jumps. Based on the rate constants from the Eyring equation, half‐life periods (t_1/2_) for the adsorption are calculated assuming a first order behavior.

	Zeolite // MACE			
	CFI	BEA	MOR	IFR
*∆A^‡^*_A→B_ (kJ/mol)	21.0	64.1	103.1	92.5
*∆A^‡^*_B→A_ (kJ/mol)	20.7	41.3	72.8	84.8
*∆A^‡^*_A↔B_ (kJ/mol)	20.9	52.7	87.9	88.6
*D*_A→B_ (m^2^/s)	1.6 × 10^−10^	1.3 × 10^–^^17^	2.0 × 10^–^^24^	3.8 × 10^–^^23^
*D*_B→A_ (m^2^/s)	1.8 × 10^−10^	1.3 × 10^−13^	3.9 × 10^−19^	8.5 × 10^−22^
*D*_A↔B_ (m^2^/s)	1.7 × 10^−10^	1.3 × 10^−15^	8.8 × 10^−22^	1.8 × 10^−22^
*t*_1/2,A→B_ (h)	1.4 × 10^−13^	4.5 × 10^−6^	27.6	0.4
*t*_1/2,B→A_ (h)	1.3 × 10^−13^	4.7 × 10^−10^	1.4 × 10^−4^	0.02
*t*_1/2,A↔B_ (h)	1.3 × 10^−13^	4.6 × 10^−8^	0.06	0.08

For the small activation barrier in CFI, very short half‐life periods are calculated, indicating an instantaneous adsorption. In MOR, transition state theory connects the barrier *∆A^‡^*_A→B_ of 103.1 kJ/mol to a half‐life on the order of hours for each diffusion jump, making the process hardly observable on typical laboratory time scales. This presents an apparent contradiction with experimental evidence showing significant uptake of CBZ into MOR within 24 h [16]. The half‐life calculated from the *∆A^‡^*_B→A_ barrier or from the mean of the forward and the backward jump (*∆A^‡^*_A↔B_) results in a value which is more in line with experimental evidence of adsorption within laboratory time scales. In addition to that, by considering the complete, multi‐step adsorption process from the aqueous phase, which is governed by more than just the intra‐crystalline barrier, this can be further resolved and explained.

**FIGURE 5:**
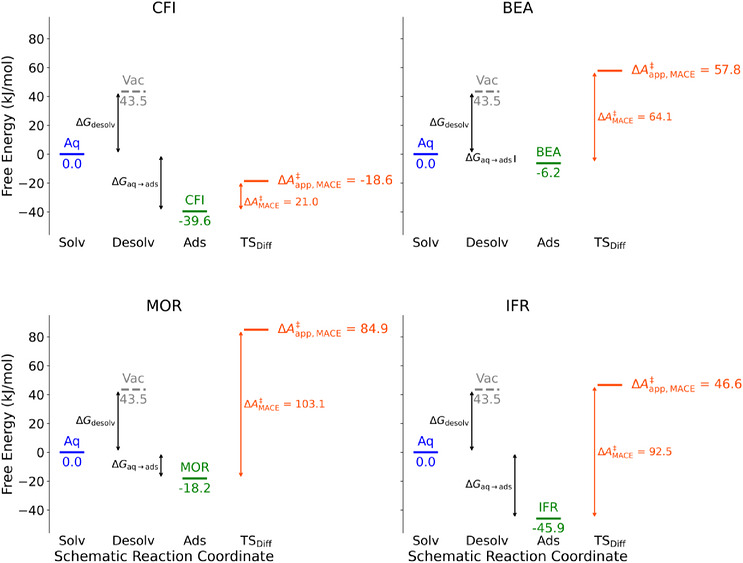
Schematic adsorption process of CBZ from water into the zeolite with the associated free energy values in kJ/mol. TS_Diff_ denotes the diffusion transition state of the respective system. The activation free energies (∆*A* ^‡^) can also be found in Table 1. All energies are referenced to the free solvation energy of ‐43.46 kJ/mol, thus at TS_Diff_ the apparent activation energies (∆*A* ^‡^_app_) are printed. The MACE transition states are depicted in orange, the adsorption free energies are depicted in green.

The experimental rate of uptake is influenced by several factors not accounted for in a single‐molecule simulation such as the thermodynamic driving force from the chemical potential gradient at finite concentrations and the energetic cost of desolvation, as well as initial surface adsorption and cooperative multi‐molecule effects. From FEP simulations employing the SPC/fw water model, a free solvation energy of ‐43.5 kJ/mol for CBZ is computed, which directly corresponds to the negative of the desolvation energy. To visualize the interplay of these energetic contributions and obtain a comparative picture of the relevant energetic states, Figure 5 shows a schematic energy diagram of the adsorption from water. In each case the diffusion barrier *∆A^‡^*_A→B_ is taken as the respective diffusion barrier and all energies are referenced to the free solvation energy of CBZ, thus the energy values at TS_Diff_ correspond to apparent activation energy *∆A^‡^*_app_ [62].

Upon adsorption, which is exergonic for all depicted systems, the system ends up with excess energy, which could be translated into kinetic energy, decreasing the diffusion barrier to the apparent activation energy *∆A^‡^*_app_ shown in the energy diagrams. For CFI (Figure 5, top left) this makes even the diffusion transition states energetically favorable compared to the solvated state state, which could indicate that for such extra‐large pore zeolites desolvation could be the rate‐determining step. For the BEA framework the barrier is not significantly lowered, because of the small adsorption free energy in this framework, while in MOR and IFR the apparent activation energy is substantially lowered to 84.9 kJ/mol and 46.6 kJ/mol respectively. For MOR, this would correspond to a half‐life of about 1 min, instead of 27.6 h, which might be more in line with the experimentally observed adsorption of CBZ by MOR.

This demonstrates that the interplay between solvation, adsorption, and diffusion is a complex multifactor process, and assessing the individual contributions is essential to understand the complete adsorption process. While a direct comparison between a kinetic diffusion barrier and a thermodynamic state difference (Δ*G*_desolv_) is a simplification, their relative magnitudes still provide insight into the likely rate‐determining step of the overall process.

## 4 Summary and Outlook

This work presents a comprehensive computational study on the diffusion of the persistent pharmaceutical pollutant CBZ in a range of all‐silica zeolite frameworks. By employing both classical force fields and neural network potentials (MACE‐based) in umbrella sampling simulations, we moved beyond static adsorption energies and assess the kinetic barriers governing the efficacy of zeolites as adsorbents. The umbrella sampling simulations were optimized in terms of the chosen force constant and the spacing of the umbrella windows based on the OGRe protocol [57].

Initial long‐timescale equilibrium classical MD simulations confirmed that while CBZ is mobile in extra‐large pore zeolites, its diffusion cannot be sufficiently sampled using these simulations in frameworks with tighter pores such as IFR, necessitating the use of enhanced sampling methods. Umbrella sampling simulations at the classical FF level identified a clear distinction between low‐barrier diffusion in most extra‐large pore systems and FAU and distinct jump‐diffusion behavior in large‐pore zeolites like IFR, MOR, and BEA and the extra‐large pore framework CFI.

The MACE simulations showed significant, system‐dependent deviations with respect to the classical FF picture in the more confining zeolite pores (BEA, MOR, and IFR), while being in quantitative agreement in the less narrow extra‐large pore zeolite CFI, in which CBZ occupies mostly close‐to‐equilibrium structures. Despite these quantitative deviations, the overall shape of the FES showed good qualitative agreement between the classical FF and MACE descriptions for all systems. We report that the chosen classical force field parameters were able to accurately represent diffusion in a close‐to‐equilibrium‐regime, while in the more strained configurations that occur in the narrower pores of BEA, IFR, and MOR, significant deviations from the MACE description emerged. Another critical insight provided by the MACE simulations is the identification of slow relaxation. After overcoming a diffusion barrier, the CBZ molecule frequently populates higher‐energy, metastable orientations, leading to a pronounced asymmetry in the calculated FES, which decreased with elongated simulations times or an improved equilibration strategy. This can be understood as a physically meaningful consequence of the complex quantum‐mechanical energy landscape, where significant kinetic barriers exist for local molecular re‐orientation and framework relaxation, which can be slow processes. Additionally, by also considering the CBZ molecule in the solvated state, the apparent diffusion barrier decreases significantly, since the adsorption from water is exergonic in all large and extra‐large pore zeolites considered.

It is worth noting that the evaluation of the bias‐corrected potential energies of the systems on the classical FF and MACE level of theory (section S5) showed that the MACE simulations were consistently more robust in finding a stable minimum of the zeolite structures, as they only showed local distortions  after diffusion jumps.

This finding has direct and important implications for interpreting the diffusion process in a real‐world context. While the calculated activation barrier from the global minimum might be high in some cases, the initial adsorption from an aqueous phase is exergonic and the excess energy can be converted into kinetic energy or to populate higher‐energy metastable states. Both cases would be reflected in a lowering of the activation barrier and an increased self‐diffusion coefficient. By considering the apparent activation energy for CBZ in the MOR framework, we arrive at a half‐life that is consistent with experimental reports of an equilibrated adsorption after 24 h [16].

In conclusion, this work demonstrates that the diffusion of CBZ in shape‐selective zeolites is governed by a complex interplay between translational and rotational energy barriers and slow relaxation after diffusion events. This refined understanding opens new approaches to the rational design of novel adsorbents. Our central conclusion, that a strong, shape‐selective interaction does not necessarily prohibit diffusion, is particularly encouraging. However, the significant influence of kinetic trapping suggests that the goal of materials design should focus on engineering a free energy landscape with a deep and kinetically accessible minimum to ensure both high capacity and efficient transport kinetics.

This work also highlights the critical role of state‐of‐the‐art machine‐learned potentials in describing such detailed dynamic behavior. Future computational efforts should aim to explicitly model the adsorption from an aqueous phase to validate our hypothesis regarding the population of metastable states upon desolvation and to obtain dynamic insight into the desolvation process. Extending this validated workflow to investigate a wider range of persistent organic pollutants could accelerate the discovery of optimal zeolite candidates for targeted environmental remediation.

## Author Contributions

Investigation and Formal analysis were done by **Jakob Brauer**. **Richard Kendra** supplied the automated OGRe workflow to the Methodology, which was slightly adapted by **Jakob Brauer**. **Carlos Bornes** and **Lukáš Grajciar** supported the development of the fine‐tuned MACE potential. Writing of the original draft, rewriting and visualization were done by **Jakob Brauer**. All authors were involved in the discussion on the analysis and the results as well as the review and editing of the draft manuscript. Supervision was done by **Michael Fischer**.

## Conflicts of Interest

The authors declare no conflicts of interest.

## Supporting information



The supporting information to this work contains a thorough validation of the finetuned MACE potential alongside with a description of the data used for the fine tuning in section S1. Section S2 shows a validation of the umbrella sampling approach. In section S3 the absolute displacement of the CBZ molecule during the equilibrium MD simulations, calculated from the MSD is shown. Section S4 contains an in‐depth discussion of the asymmetric FES on the classical FF level of theory, section S5 compares the classical FF and MACE FES and assesses the effect of longer data production and high‐temperature equilibration to enhance the sampling. The Supporting Information Files archive contains datafiles, inputs, scripts and cif files, to reproduce the umbrella sampling simulations as well as the raw data of the FES alongside with the OGRe metrics of each free energy surface and the Time‐evolution_PE.xslx file containing the potential energies of the respective configurations. This file is available from: https://doi.org/10.26434/chemrxiv‐2025‐7×5nf.

## References

[1] S. J. Nevitt , A. G. Marson , and C. T. Smith , “Carbamazepine Versus Phenytoin Monotherapy for Epilepsy: An Individual Participant Data Review,” Cochrane Database of Systematic Reviews 2019 (2019), 10.1002/14651858.CD001911.pub4. PMC6637502 31318037

[2] S. J. Nevitt , A. G. Marson , J. Weston , and C. T. Smith , “Sodium Valproate Versus Phenytoin Monotherapy for Epilepsy: An Individual Participant Data Review,” Cochrane Database of Systematic Reviews 2018 (2018), 10.1002/14651858.CD001769.pub4. PMC6513104 30091458

[3] “ClinCalc Carbamazepine Drug Usage Statistics, Unites States, 2014–2023,” can be found under https://clincalc.com/drugstats/Drugs/Carbamazepine(accessed 7 July 2025), 2025.

[4] R. Oldenkamp , A. H. W. Beusen , and M. A. J. Huijbregts , “Aquatic Risks from human Pharmaceuticals—modelling Temporal Trends of Carbamazepine and Ciprofloxacin at the Global Scale,” Environmental Research Letters 14 (2019): 034003.

[5] M. Clara , B. Strenn , and N. Kreuzinger , “Carbamazepine as a Possible Anthropogenic Marker in the Aquatic Environment: Investigations on the Behaviour of Carbamazepine in Wastewater Treatment and during Groundwater Infiltration,” Water Research 38 (2004): 947–954. 14769414 10.1016/j.watres.2003.10.058

[6] H. Zind , L. Mondamert , Q. B. Remaury , A. Cleon , N. K. V. Leitner , and J. Labanowski , “Occurrence of Carbamazepine, Diclofenac, and Their Related Metabolites and Transformation Products in a French Aquatic Environment and Preliminary Risk Assessment,” Water Research 196 (2021): 117052. 33774347 10.1016/j.watres.2021.117052

[7] Y. Zhang , S.‐U. Geißen , and C. Gal , “Carbamazepine and Diclofenac: Removal in Wastewater Treatment Plants and Occurrence in Water Bodies,” Chemosphere 73 (2008): 1151–1161. 18793791 10.1016/j.chemosphere.2008.07.086

[8] Â. Almeida , A. M. V. M. Soares , V. I. Esteves , and R. Freitas , “Occurrence of the Antiepileptic Carbamazepine in Water and Bivalves From Marine Environments: A Review,” Environmental Toxicology and Pharmacology 86 (2021): 103661. 33878451 10.1016/j.etap.2021.103661

[9] N. S. P. Batucan , L. A. Tremblay , G. L. Northcott , and C. D. Matthaei , “Medicating the Environment? A Critical Review on the Risks of Carbamazepine, Diclofenac and Ibuprofen to Aquatic Organisms,” Environmental Advances 7 (2022): 100164, 10.1016/j.envadv.2021.100164.

[10] E. Donner , T. Kosjek , S. Qualmann , et al., “Ecotoxicity of Carbamazepine and Its UV Photolysis Transformation Products,” Science of the Total Environment 443 (2013): 870–876. 23247289 10.1016/j.scitotenv.2012.11.059

[11] L. Qiang , J. Cheng , J. Yi , J. M. Rotchell , X. Zhu , and J. Zhou , “Environmental Concentration of Carbamazepine Accelerates Fish Embryonic Development and Disturbs Larvae Behavior,” Ecotoxicology (London, England) 25 (2016): 1426–1437. 27386877 10.1007/s10646-016-1694-y

[12] V. L. Cunningham , C. Perino , V. J. D'Aco , A. Hartmann , and R. Bechter , “Human Health Risk Assessment of Carbamazepine in Surface Waters of North America and Europe,” Regulatory Toxicology and Pharmacology 56 (2010): 343–351. 19883710 10.1016/j.yrtph.2009.10.006

[13] E. Kaiser , C. Prasse , M. Wagner , K. Bröder , and T. A. Ternes , “Transformation of Oxcarbazepine and Human Metabolites of Carbamazepine and Oxcarbazepine in Wastewater Treatment and Sand Filters,” Environmental Science & Technology 48 (2014): 10208–10216. 25137395 10.1021/es5024493

[14] D. J. De Ridder , A. R. D. Verliefde , S. G. J. Heijman , et al., “Influence of Natural Organic Matter on Equilibrium Adsorption of Neutral and Charged Pharmaceuticals onto Activated Carbon,” Water Science and Technology 63 (2011): 416–423. 21278462 10.2166/wst.2011.237

[15] A. Rossner , S. A. Snyder , and D. R. U. Knappe , “Removal of Emerging Contaminants of Concern by Alternative Adsorbents,” Water Research 43 (2009): 3787–3796. 19577267 10.1016/j.watres.2009.06.009

[16] D. J. De Ridder , J. Q. J. C. Verberk , S. G. J. Heijman , G. L. Amy , and J. C. Van Dijk , “Zeolites for Nitrosamine and Pharmaceutical Removal From Demineralised and Surface Water: Mechanisms and Efficacy,” Separation and Purification Technology 89 (2012): 71–77.

[17] A. Georgi , R. Köhler , S. Woszidlo , et al., “Fe‐Zeolite as on‐Site Regenerable Adsorber for Chlorohydrocarbons in Groundwater—From Laboratory to Pilot Test,” Chemie Ingenieur Technik 95 (2023): 1999–2007.

[18] S. Kulprathipanja and R. B. James , “Overview in Zeolites Adsorptive Separation,” in: Zeolites in Industrial Separation and Catalysis (Wiley, 2010), 173–202.

[19] C. P. Nicholas , “Overview and Recent Developments in Catalytic Applications of Zeolites,” in: Zeolites in Industrial Separation and Catalysis (Wiley, 2010), 355–402.

[20] A. Martucci , L. Pasti , N. Marchetti , A. Cavazzini , F. Dondi , and A. Alberti , “Adsorption of Pharmaceuticals From Aqueous Solutions on Synthetic Zeolites,” Microporous and Mesoporous Materials 148 (2012): 174–183.

[21] W. A. Cabrera‐Lafaurie , F. R. Román , and A. J. Hernández‐Maldonado , “Removal of Salicylic Acid and Carbamazepine From Aqueous Solution With Y‐Zeolites Modified With Extraframework Transition Metal and Surfactant Cations: Equilibrium and Fixed‐Bed Adsorption,” Journal of Environmental Chemical Engineering 2 (2014): 899–906.

[22] O. A. Al‐Mashaqbeh , D. A. Alsafadi , L. Z. Alsalhi , S. L. Bartelt‐Hunt , and D. D. Snow , “Removal of Carbamazepine onto Modified Zeolitic Tuff in Different Water Matrices: Batch and Continuous Flow Experiments,” Water (Basel) 13 (2021): 1084.

[23] M. Fischer , “Simulation‐based Evaluation of Zeolite Adsorbents for the Removal of Emerging Contaminants,” Materials Advances 1 (2020): 86–98.

[24] J. Brauer and M. Fischer , “Computational Screening of Hydrophobic Zeolites for the Removal of Emerging Organic Contaminants From Water,” Chemphyschem 25 (2024): e202400347. 38861113 10.1002/cphc.202400347

[25] M. Fischer , “Adsorption of Carbamazepine in all‐Silica Zeolites Studied With Density Functional Theory Calculations,” Chemphyschem 24 (2023): e202300022. 36715697 10.1002/cphc.202300022

[26] J. Kärger and H. Pfeifer , “N.m.r. self‐diffusion Studies in Zeolite Science and Technology,” Zeolites 7 (1987): 90–107.

[27] J. Kärger , M. Avramovska , D. Freude , J. Haase , S. Hwang , and R. Valiullin , “Pulsed Field Gradient NMR Diffusion Measurement in Nanoporous Materials,” Adsorption 27 (2021): 453–484.

[28] C. Hernandez‐Tamargo , I. P. Silverwood , A. J. O'Malley , and N. H. de Leeuw , “Quasielastic Neutron Scattering and Molecular Dynamics Simulation Study on the Molecular Behaviour of Catechol in Zeolite Beta,” Topics in Catalysis 64 (2021): 707–721.

[29] J. J. Erik Maris , L. A. Parker , K. Stanciakova , et al., “Molecular Accessibility and Diffusion of Resorufin in Zeolite Crystals,” Chemistry—A European Journal 30 (2024): e202302553. 37815001 10.1002/chem.202302553

[30] R. Rungsirisakun , T. Nanok , M. Probst , and J. Limtrakul , “Adsorption and Diffusion of Benzene in the Nanoporous Catalysts FAU, ZSM‐5 and MCM‐22: a Molecular Dynamics Study,” Journal of Molecular Graphics and Modelling 24 (2006): 373–382. 16288979 10.1016/j.jmgm.2005.10.003

[31] D. Zhai , L. Zhao , J. Gao , and C. Xu , “Effect of Temperature on the Diffusion Mechanism of Xylene Isomers in a FAU Zeolite: A Molecular Dynamics Study,” Physical Chemistry Chemical Physics 14 (2012): 7296. 22531835 10.1039/c2cp40584a

[32] M. Khalkhali , A. Ghorbani , and B. Bayati , “Study of Adsorption and Diffusion of Methyl Mercaptan and Methane on FAU Zeolite Using Molecular Simulation,” Polyhedron 171 (2019): 403–410.

[33] M. P. Allen and D. J. Tildesley , “Introduction,” in: Computer Simulation of Liquids (Oxford University PressOxford, 2017), 1–45.

[34] M. DeLuca and D. Hibbitts , “Predicting Diffusion Barriers and Diffusivities of C6–C12 Methylbenzenes in MFI Zeolites,” Microporous and Mesoporous Materials 333 (2022): 111705.

[35] G. M. Torrie and J. P. Valleau , “Nonphysical Sampling Distributions in Monte Carlo Free‐Energy Estimation: Umbrella Sampling,” Journal of Computational Physics 23 (1977): 187–199.

[36] P. Pongprayoon , O. Beckstein , C. L. Wee , and M. S. P. Sansom , “Simulations of Anion Transport through OprP Reveal the Molecular Basis for High Affinity and Selectivity for Phosphate,” Proceedings of the National Academy of Sciences 106 (2009): 21614–21618. 10.1073/pnas.0907315106 PMC2789165 19966228

[37] K. Atkovska and J. S. Hub , “Energetics and Mechanism of Anion Permeation across Formate‐nitrite Transporters,” Scientific Reports 7 (2017): 12027. 28931899 10.1038/s41598-017-11437-0 PMC5607303

[38] M. Zhang , F. Sicard , T. S. Erkal , G. M. Bowers , and A. O. Yazaydin , “The Role of Surface Thermodynamics and Kinetics in the Removal of PFOA from Aqueous Solutions,” Surfaces and Interfaces 41 (2023): 103271.

[39] J. Zhou , G. Cui , S. Hu , et al., “Graph Neural Networks: a Review of Methods and Applications,” AI Open 1 (2020): 57–81.

[40] F. Jensen , Introduction to Computational Chemistry (Wiley Inc., 2006).

[41] I. Batatia , D. P. Kovacs , G. Simm , C. Ortner , and G. Csanyi in Advances in Neural Information Processing Systems (Eds.: S. Koyejo , S. Mohamed , S. Agarwal , D. Belgrave , K. Cho , A. Oh ), Curran Associates, Inc., vol. 35, 2022: 11423–11436.

[42] I. Batatia , P. Benner , Y. Chiang , et al., “A Foundation Model for Atomistic Materials Chemistry,” The Journal of Chemical Physics 163 (2025): 184110. 41230846 10.1063/5.0297006

[43] A. P. Thompson , H. M. Aktulga , R. Berger , et al., “LAMMPS—A Flexible Simulation Tool for Particle‐Based Materials Modeling at the Atomic, Meso, and Continuum Scales,” Computer Physics Communications 271 (2022): 108171.

[44] S. Boothroyd , P. K. Behara , O. C. Madin , et al., “Development and Benchmarking of Open Force Field 2.0.0: The Sage Small Molecule Force Field,” Journal of Chemical Theory and Computation 19 (2023): 3251–3275. 37167319 10.1021/acs.jctc.3c00039 PMC10269353

[45] F. S. Emami , V. Puddu , R. J. Berry , et al., “Force Field and a Surface Model Database for Silica to Simulate Interfacial Properties in Atomic Resolution,” Chemistry of Materials 26 (2014): 2647–2658.

[46] J. Brauer , P. Mahdavi , J. Thöming , and M. Fischer , “Understanding the Adsorption Trilemma: Achieving Load, Level, and Selectivity for the Removal of Pharmaceuticals with Zeolites,” Separation and Purification Technology 392 (2026): 137086.

[47] S. Nosé , “A Unified Formulation of the Constant Temperature Molecular Dynamics Methods,” Journal of Chemical Physics 81 (1984): 511–519.

[48] W. G. Hoover , “Canonical Dynamics: Equilibrium Phase‐Space Distributions,” Physical Review A (Coll Park) 31 (1985): 1695–1697. 10.1103/physreva.31.1695 9895674

[49] R. W. Hockney and J. Eastwood , Computer Simulation Using Particles (Hilger, Bristol, 1988).

[50] J.‐P. Ryckaert , G. Ciccotti , and H. J. C. Berendsen , “Numerical Integration of the Cartesian Equations of Motion of a System With Constraints: Molecular Dynamics of n‐Alkanes,” Journal of Computational Physics 23 (1977): 327–341.

[51] A. Hjorth Larsen , J. Jørgen Mortensen , J. Blomqvist , et al., “The Atomic Simulation Environment—A Python Library for Working With Atoms,” Journal of Physics: Condensed Matter 29 (2017): 273002. 28323250 10.1088/1361-648X/aa680e

[52] The PLUMED consortium , “Promoting Transparency and Reproducibility in Enhanced Molecular Simulations,” Nature Methods 16 (2019): 670–673. 31363226 10.1038/s41592-019-0506-8

[53] H. Grubmüller , B. Heymann , and P. Tavan , “Ligand Binding: Molecular Mechanics Calculation of the Streptavidin‐Biotin Rupture Force,” Science 271 (1979): 997–999. 10.1126/science.271.5251.997 8584939

[54] M. R. Shirts and J. D. Chodera , “Statistically Optimal Analysis of Samples From Multiple Equilibrium States,” Journal of Chemical Physics 129 (2008): 124105. 19045004 10.1063/1.2978177 PMC2671659

[55] M. R. Shirts and A. L. Ferguson , “Statistically Optimal Continuous Free Energy Surfaces from Biased Simulations and Multistate Reweighting,” Journal of Chemical Theory and Computation 16 (2020): 4107–4125. 32497425 10.1021/acs.jctc.0c00077

[56] C. H. Bennett , “Efficient Estimation of Free Energy Differences From Monte Carlo Data,” Journal of Computational Physics 22 (1976): 245–268.

[57] S. Borgmans , S. M. J. Rogge , L. Vanduyfhuys , and V. Van Speybroeck , “OGRe: Optimal Grid Refinement Protocol for Accurate Free Energy Surfaces and Its Application in Proton Hopping in Zeolites and 2D COF Stacking,” Journal of Chemical Theory and Computation 19 (2023): 9032–9048. 38084847 10.1021/acs.jctc.3c01028 PMC10753773

[58] S. Kumar , J. M. Rosenberg , D. Bouzida , R. H. Swendsen , and P. A. Kollman , “The Weighted Histogram Analysis Method for Free‐Energy Calculations on Biomolecules. I. The Method,” Journal of Computational Chemistry 13 (1992): 1011–1021.

[59] J. C. B. Dietschreit , D. J. Diestler , A. Hulm , C. Ochsenfeld , and R. Gómez‐Bombarelli , “From Free‐Energy Profiles to Activation Free Energies,” Journal of Chemical Physics 157 (2022): 084113. 36050004 10.1063/5.0102075

[60] M. Pinheiro , R. L. Martin , C. H. Rycroft , A. Jones , E. Iglesia , and M. Haranczyk , “Characterization and Comparison of Pore Landscapes in Crystalline Porous Materials,” J Mol Graph Model 44 (2013): 208–219. 23876827 10.1016/j.jmgm.2013.05.007

[61] P. Hänggi , P. Talkner , and M. Borkovec , “Reaction‐rate Theory: Fifty Years after Kramers,” Review of Modern Physics 62 (1990): 251–341.

[62] E. Roduner , “Understanding Catalysis,” Chemical Society Reviews 43 (2014): 8226–8239. 25311156 10.1039/c4cs00210e

