# Supplementary material for: Diffusion of Carbamazepine in Hydrophobic Zeolites: A Comparative Study Using Classical and Machine‐Learned Potentials
Source: Chemistry. 2026 Apr 24;32(26):e71048. doi: 10.1002/chem.71048 (PMC13356378; doi:10.1002/chem.71048)
Supplement: Supplementary file 1 — The supporting information to this work contains a thorough validation of the finetuned MACE potential alongside with a description of the data used for the fine tuning in section S1. Section S2 shows a validation of the umbrella sampling approach. In section S3 the absolute displacement of the CBZ molecule during the equilibrium MD simulations, calculated from the MSD is shown. Section S4 contains an in‐depth discussion of the asymmetric FES on the classical FF level of theory, section S5 compares the classical FF and MACE FES and assesses the effect of longer data production and high‐temperature equilibration to enhance the sampling. The Supporting Information Files archive contains datafiles, inputs, scripts and cif files, to reproduce the umbrella sampling simulations as well as the raw data of the FES alongside with the OGRe metrics of each free energy surface and the Time‐evolution_PE.xslx file containing the potential energies of the respective configurations. This file is available from: https://doi.org/10.26434/chemrxiv‐2025‐7×5nf. [file CHEM-32-e71048-s001.pdf]

Diffusion of Carbamazepine in Hydrophobic Zeolites:

A Comparative Study Using Classical and Machine-Learned Potentials

SUPPORTING INFORMATION

Jakob Brauer*[a,b], Richard Kendra[c], Carlos Bornes[c], Lukáš Grajciar[c], Michael Fischer*[a,b]

[a] Crystalline Microporous Materials, Crystallography and Geomaterials Research, Faculty of Geosciences, University of Bremen, Klagenfurter Straße 2 4, 28359 Bremen, Germany

[b] Bremen Center for Computational Materials Science and MAPEX Center for Materials and Processes, University of Bremen, 28359 Bremen, Germany

[c] Department of Physical and Macromolecular Chemistry, Charles University, Hlavova 8, 12800 Praha 2, Czech Republic

* Corresponding authors: E-Mail: jabr@uni-bremen.de, michael.fischer@uni-bremen.de

ORCID: 0000-0001-5133-1537 (Michael Fischer)

0009-0007-6578-4802 (Jakob Brauer)

S1. Validation of the finetuned MACE potential

The DFT reference data was calculated using the Quickstep module of the CP2K software.[1] All calculations employed the rev-vdW-DF2 functional[2] and GTH pseudopotentials[3]. AIMD simulations were done using DZVP-MOLOPT-SR basis sets with a plane wave cutoff of 600 Ry and a dispersion cutoff of 300 Ry in the *NVT* ensemble at 573 K. Subsampled snapshots for training of the MACE potential were recalculated using TZVP-MOLOPT basis sets[4] with a plane wave cutoff of 900 Ry and a dispersion cutoff of 600 Ry.

The configurations were subsampled by farthest point sampling (FPS) on the MACE atomic energies of the configurations. Subsampling was done using the fpsample module with the FPS+NPDU(nearest-point-distance-updating[5])+KDTree method. From the AIMD data, 200 configurations of each zeolite were obtained from subsampling for the first training iteration.

The MACE foundation model MACE-MP-0b2[6] (with L=1, medium size) was iteratively trained, first finetuning with TZVP recalculated snapshots taken from the AIMD DZVP trajectories at 573 K of CBZ in the zeolite frameworks CFI, FAU, MOR, BEA and IFR. With this first finetuned model additional simulations were carried out: i) high temperature MD simulations at 1000 K of the adsorbed and isolated systems (bare zeolites and CBZ), ii) isotropic zeolite cell deformations in 1% steps ranging from 95% to 105% of the initial cell dimensions, and iii) nudged-elastic-band calculations of the CBZ diffusion, ran with the janus-core software.[7] The high temperature data was subsampled in the same fashion as the AIMD data. All new configurations were recalculated on DFT level in line with the methodology described above and included in the dataset for the finetuning of the final MACE potential, which was then retrained again. The following figures show the validation of the final finetuned model against DFT reference data on the NEB images and interaction energies of the low-energy configurations. For completeness and comparison, the performance of the foundation model and a model trained from scratch in the same fashion as the foundation model (only using the finetuning data) is included in the validation.

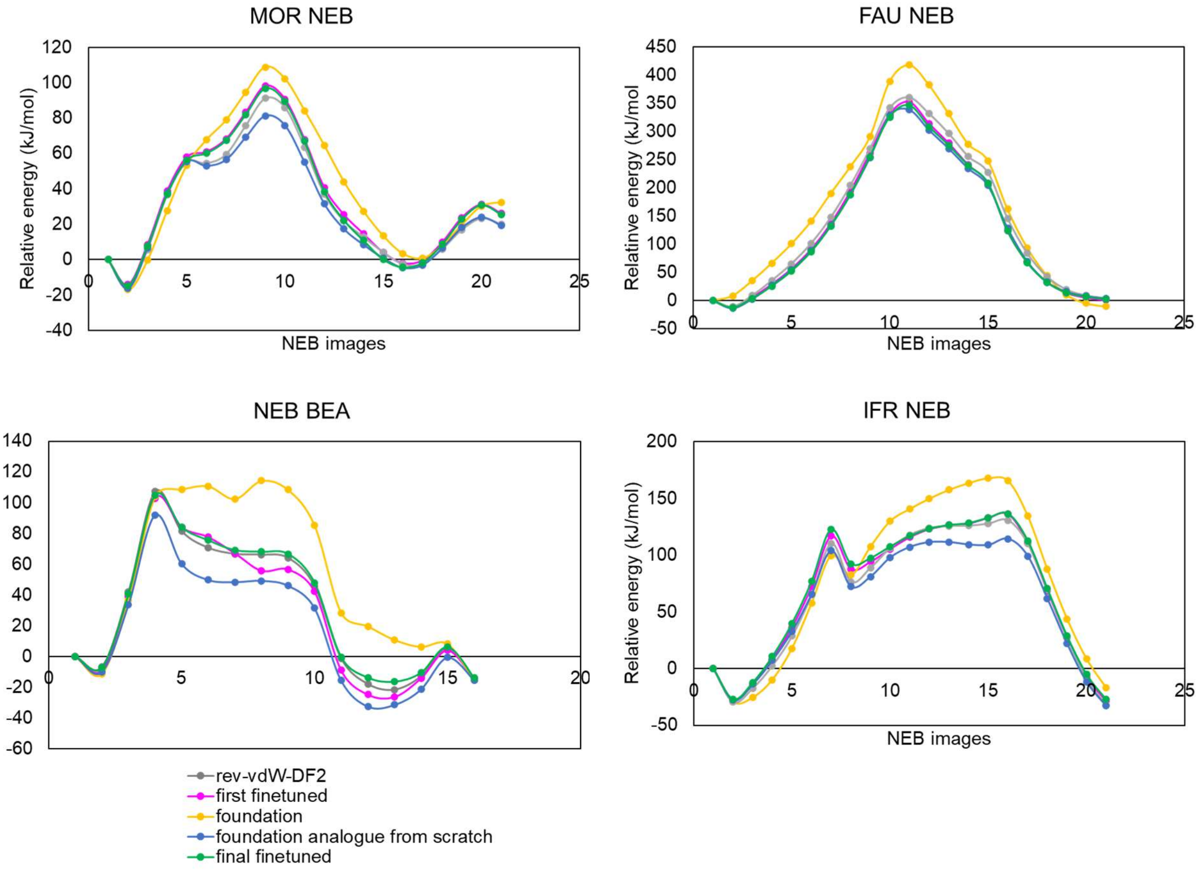

Figure S 1: NEB scans of CBZ in the respective zeolite framework shown for the reference data (grey), the foundation model (orange), the model trained from scratch (blue), the first finetuned model (magenta) and the final finetuned model (green).

The NEB scans in Figure S 1 show that the foundation model provides a good starting point for the investigation but needs to be improved as it overestimates the energies in the high-energy regime, which is governed by close atomic contacts and significant deformations of the zeolite. Even after the first finetuning, using only the AIMD data the overlap improves strongly, the main deviation can be seen for the NEB in BEA. The final finetuned model leads to an excellent overlap between the reference data and the model. Training a model from scratch with the same data and the same architecture as the medium_mp0_b2 foundation model shows a good qualitative agreement between the reference data and the MACE potential, but a constant underestimation of the high-energy regime, making the finetuned model the best choice to model the diffusion with molecular dynamics and umbrella sampling.

Figure S 2 shows the interaction energies of CBZ with the respective zeolite frameworks in the respective low-energy configurations. The interaction energies are computed on the same structures with the first finetuned model, the final finetuned model, the model trained from scratch and the foundation model in combination with the D3 correction.

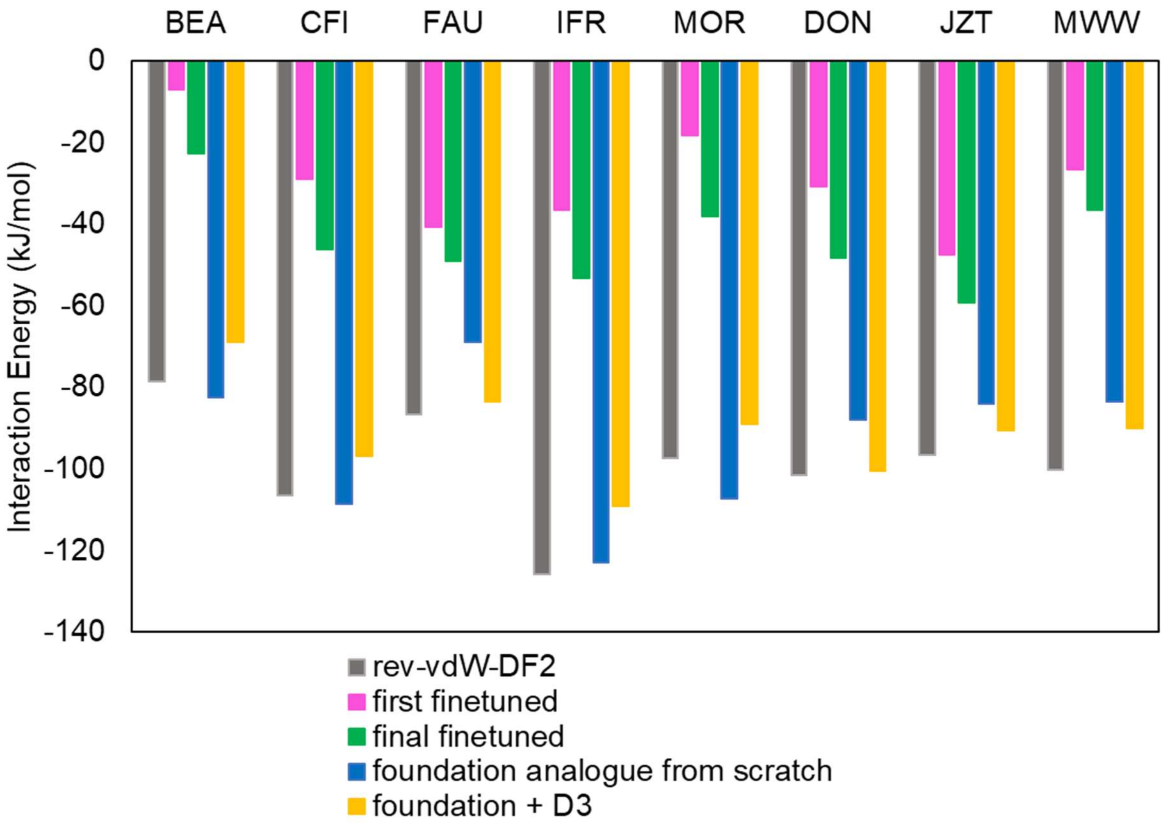

Figure S 2: Interaction energies of CBZ in the respective zeolite framework shown for the reference data (grey), the foundation model (yellow), the model trained from scratch analogue to the foundation model (blue) and the first (pink) and the final finetuned model (green).

A clear underestimation of the interaction energies for both finetuned models is apparent. The first finetuned model (pink) used only data from AIMD trajectories of the adsorbed systems, so it lacked information on the isolated systems, which is slightly improved for the final finetuned model but still strongly and unsystematically underestimated. The model trained from scratch with only the rev-vdW-DF2 data and the foundation model both perform reasonably well on the interaction energies, both with a slight tendency to underestimate the interaction energy, which can be attributed to the total receptive field of an atom of 10.0 Å, which is arguably smaller than in the DFT treatment of the systems.

In addition to the NEB scans and the DFT adsorption configurations, we also tested the performance of the classical FF parameters and the MACE foundation model and the final finetuned model against DFT interaction energies (see Table S 1). The values deviate from the ones plotted in Figure S2 since those configurations were DFT geometries, while here we consider optimized classical FF configurations. The same trend we already described in our previous work holds, the classical FF parameters systematically underestimate the interaction energy. The foundation model without the D3 correction yields strongly positive interaction energies, indicating that the Pauli-repulsion is properly learned from the MP-dataset, but no dispersion is learned, since the MP-dataset should only contain GGA data. This is particularly

prominent for the case of FAU and JZT, the two frameworks with the widest cages/channels and lowest framework density, where the limited amount of close atomic contacts gives very little Pauli-repulsion. Adding the D3 correction to the foundation model (as intended) gives proper interaction energies with a similar agreement to the classical FF values.

Considering the finetuned potential without the D3, again a strong underestimation is observed. As already stated in the previous reply, we attribute this to the attempt of the finetuned potential to learn dispersion from the rev-vdW-DF2 data, while the replay dataset counteracts this. Adding the D3 correction to the finetuned potential leads to a strong overestimation, since the partially learned dispersion is then overcorrected by the D3 correction.

Table S 1: Interaction energies of classical FF configurations calculated with rev-vdW-DF2 and MACE models with (w\) and without (w\o) D3 dispersion correction.

CBZ in ZEO (kJ/mol)	Classical FF	rev-vdW-DF2	MACE Foundation w\o D3	MACE Foundation w\ D3	MACE Final finetuned w\o D3	MACE Final Finetuned w\ D3
BEA	-99.40	-105.91	96.95	-95.46	-43.15	-235.56
CFI	-120.39	-133.82	61.71	-123.24	-65.26	-250.21
FAU	-103.12	-113.98	11.29	-110.05	-75.06	-196.40
IFR	-130.36	-153.01	63.28	-135.69	-73.49	-272.46
MOR	-107.26	-124.58	91.22	-115.58	-57.33	-264.13
DON	-112.57	-128.67	27.20	-126.87	-65.75	-219.82
JZT	-114.66	-123.93	4.48	-117.12	-82.44	-204.03
MWW	-112.21	-127.42	45.09	-116.47	-61.59	-223.15
c Pearson	0.97		0.02	0.94	0.44	0.58

In whole, we conclude that for our envisioned application of modelling diffusion, accurately reproducing relative energies within a system is more important than the relative energies between isolated systems, which is why we chose the finetuned medium_mp0_b2 foundation model for the umbrella sampling simulations. The training data, the foundation model, the finetuned model and the script for the training is included in the ESI. The potential used for all production runs is denoted “final finetuned” in these figures.

S2. Validation of the Umbrella sampling approach

The umbrella sampling simulations were optimized iteratively according to the OGRe protocol by checking the overlap between umbrella windows and the consistency and confinement within each umbrella window and either adding windows in between to increase the overlap or increasing the force constant to improve the sampling. To validate the equilibration and production durations, the histograms of the collective variable are shown for three scenarios. A: 1 ps moving restraint from initial position of the molecule to the position of the window, 1 ps equilibration, geometry optimization, 25 ps production.

B: 1 ps moving restraint from initial position of the molecule to the position of the window, 4 ps equilibration, geometry optimization, 100 ps production.

C: 1 ps moving restraint from initial position of the molecule to the position of the window, 4 ps equilibration, geometry optimization, 1000 ps production.

	MOR	IFR
A	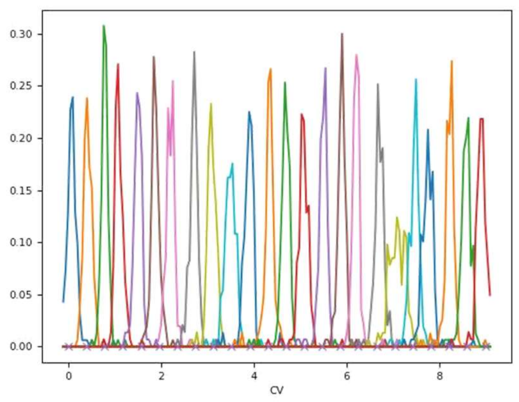	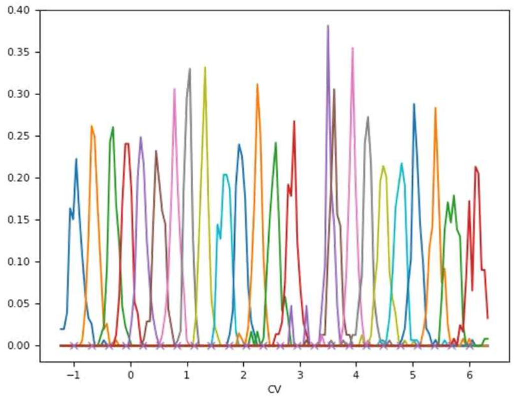
B	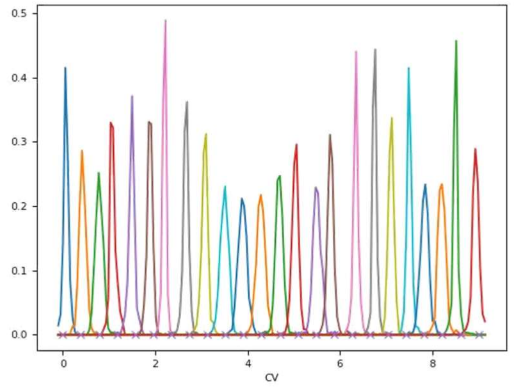	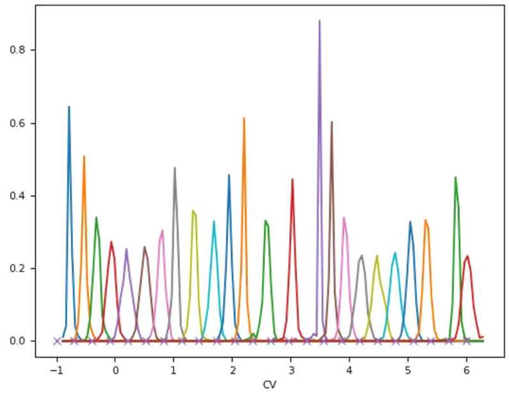

C	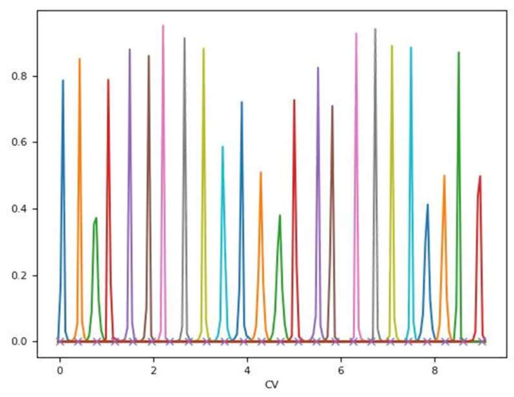	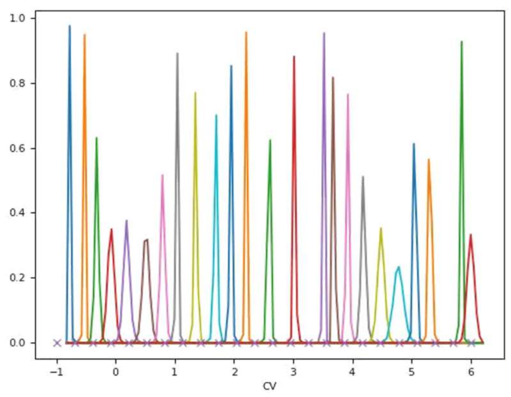

Figure S 3: Histograms of the CV at 24 equidistant positions along the CV path for three different simulation setups. A: 1 ps moving restraint from initial position of the molecule to the position of the window, 1 ps equilibration, geometry optimization, 25 ps production. B: 1 ps moving restraint from initial position of the molecule to the position of the window, 4 ps equilibration, geometry optimization, 100 ps production. C: 1 ps moving restraint from initial position of the molecule to the position of the window, 4 ps equilibration, geometry optimization, 1000 ps production. The bins were chosen at a width of 0.05 Å.

From the histograms for strategy A an imbalanced sampling due to the short equilibration time and the short data production can be observed. This is sufficiently overcome in strategy B, since the histograms shows a symmetric distribution around each point on the CV axis. Increasing the production duration by a factor of 10 consequently yields a distribution closer to the center of the CV, which improves the sampling at each position but requires more windows in between for a sufficient overlap. Since the histograms for strategy B already indicate a balanced distribution, this strategy was chosen for the umbrella sampling simulations on the classical FF level of theory and for the MACE simulations. For MOR and IFR a longer production duration in the MACE simulations is discussed in the Results and Discussion section, as well as an adaptation of the equilibration strategy for the diffusion in the IFR framework.

The Potential of mean-force (PMF) data and the OGRe metrics (overlap, confinement and consistency) for all FES shown and discussed in the manuscript is included in the ESI.

S3. Estimating the absolute displacement from the mean-squared displacement

For an intuitive measure of the distance that the CBZ molecule travelled in the unbiased simulations, the absolute distance travelled was estimated from the maximum value of the mean-squared displacement (MSD). For this, we assume a Gaussian distribution of the movement of CBZ. For isotropic 3D diffusion in interconnected frameworks such as FAU, JZT or BEA the probability *P(r,t)* of finding the CBZ molecule displaced by *r* from its origin at time *t* is given by

$$P(r,t) = \left(\frac{1}{4\pi Dt}\right)^{3/2} \exp\left(-\frac{|r|^2}{4Dt}\right) \qquad \text{(SI-Eq. 1)}$$

with *D* as the diffusion coefficient.

The MSD is then given by

$$MSD(t) = \int_0^\infty r^2 P(r,t)\, 4\pi r^2 dr \; = 4\pi \left(\frac{1}{4\pi Dt}\right)^{3/2} \int_0^\infty r^4 \exp\left(-\frac{|r|^2}{4Dt}\right) dr = 6Dt \qquad \text{(SI-Eq. 2)}$$

while the mean-absolute displacement (MAD) is

$$MAD(t) = \int_0^\infty r\, P(r,t)\, 4\pi r^2 dr \; = 4\pi \left(\frac{1}{4\pi Dt}\right)^{3/2} \int_0^\infty r^3 \exp\left(-\frac{|r|^2}{4Dt}\right) dr = 4\sqrt{\frac{Dt}{\pi}} \qquad \text{(SI-Eq. 3)}$$

and allowing to obtain the MAD as

$$MAD(t) = \frac{4}{\sqrt{6\pi}} \sqrt{MSD(t)}\,. \qquad \text{(SI-Eq. 4)}$$

The same reasoning can be applied to the 1D Gaussian displacement, which is given by

$$P(r,t) = \frac{1}{4\pi Dt} \exp\left(-\frac{r^2}{4Dt}\right). \qquad \text{(SI-Eq. 5)}$$

S4. Discussion of the asymmetric FES of CBZ diffusing through MOR, BEA and IFR calculated with classical FF

Figure S 4 shows the FES of the jump-diffusion of CBZ in IFR (green), MOR (blue) and BEA (orange) with the ensemble-averaged structures of the minima and the transition state depicted at the top. All FES do not result in the same energetic minimum the jump-diffusion started from.

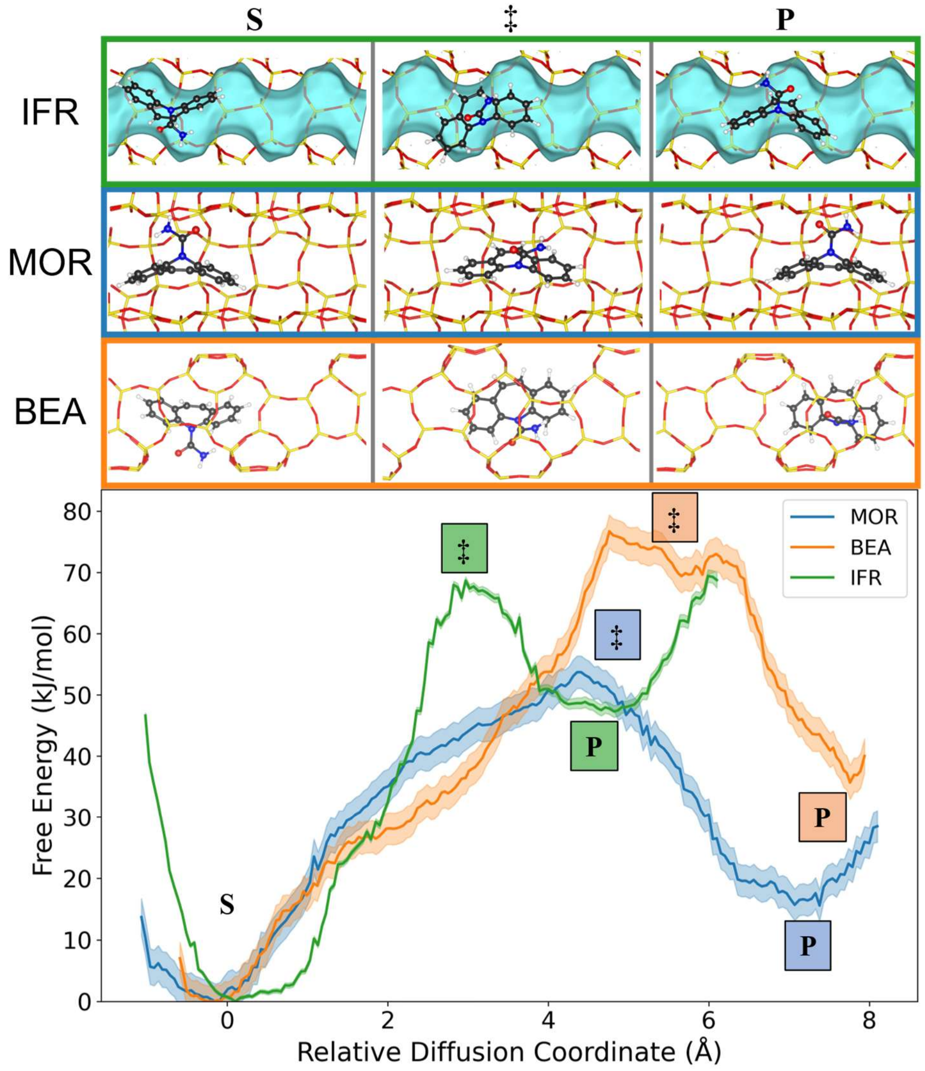

Figure S 4: FES of CBZ diffusion through MOR (blue), BEA (orange) and IFR (green). All shown FES are obtained from classical FF umbrella sampling simulations. At the top the ensemble-average structures, calculated from the full production trajectory of the corresponding umbrella windows, are shown for the initial minimum (**S**), the highest point of the barrier (‡) and the second minimum (**P**). For the IFR framework the outline of the zeolite's electron density is plotted to make the sinusoidal channels more apparent.

For the diffusion in BEA this can readily be explained from the ensemble-average configurations (Figure S 4, BEA **S**&**P** orange frame) at the initial adsorption minimum (**S**) and at the crystallographically equivalent section of the pore (**P**). CBZ starts of in its low-energy state, adsorbed in the 12 MR channel, with the amide group pointing into the perpendicularly

intersecting 12 MR channel. At the barrier, CBZ sits inside the channel between two intersections and experiences significant deformation (similar to the high-energy configuration in MOR). After that, CBZ moves further along the channel and reaches the crystallographically equivalent section of the pore at the next intersection. However, the molecule is now in a non-optimal orientation relative to the framework (see Figure S 4, BEA **S&P** orange frame), with the spatially demanding ring system spreading across the channel intersection, forcing the amide group in a less favorable configuration, pointing towards the pore wall and not into the intersection. This results in a free energy along the diffusion coordinate that is about 35 kJ/mol higher than the initial configurations. The average potential energy of the whole system (without the bias, averaged over the whole production run, plotted in Figure S 5 A or see ESI “Time-evolution_PE.xslx”) at **P** is about 50 kJ/mol higher than at **S**, supporting the conclusion, that the asymmetric FES is due to energetically different adsorption configurations at crystallographically equivalent sections of the pore.

In MOR, the profile of the FES is largely symmetrical, the minima at the crystallographically equivalent section of the pore and the initial state differ by less than 20 kJ/mol. The blue framed snapshots in Figure S 4 show CBZ in the MOR framework in the initial adsorption configuration (**S**), at the top of the barrier (‡) and in the second minimum (**P**). In the initial geometry the amide group of CBZ points upward into the 8 MR that outlines the 12 MR channel of MOR. At the energy barrier, the amide group and the annulated ring system experience a flattening deformation. After this deformation the CBZ molecule arrives at the commensurable section of the pore largely identical to the initial configuration.

The **S** and **P** configuration of the diffusion through IFR show the molecule rotating along the channels axis to fit into the next protrusion of the channel. Due to the sinusoidal channel, the molecule has to rotate along the channel axis as it moves along the pore, so the shape-topology fit can be maintained. At around 6 Å the next diffusion jump is already observable, which is energetically equivalent to the first barrier.

For both diffusion events, the rotated amide group of CBZ is unlikely to be responsible for the energetic difference between **S** and **P**. Given the symmetry of the CBZ ring system and the pore, both configurations should give equivalent energies. To understand the asymmetric FES in the case of MOR and IFR, the time-evolution of the systems bias-corrected potential energy is plotted in Figure S 5 B/C.

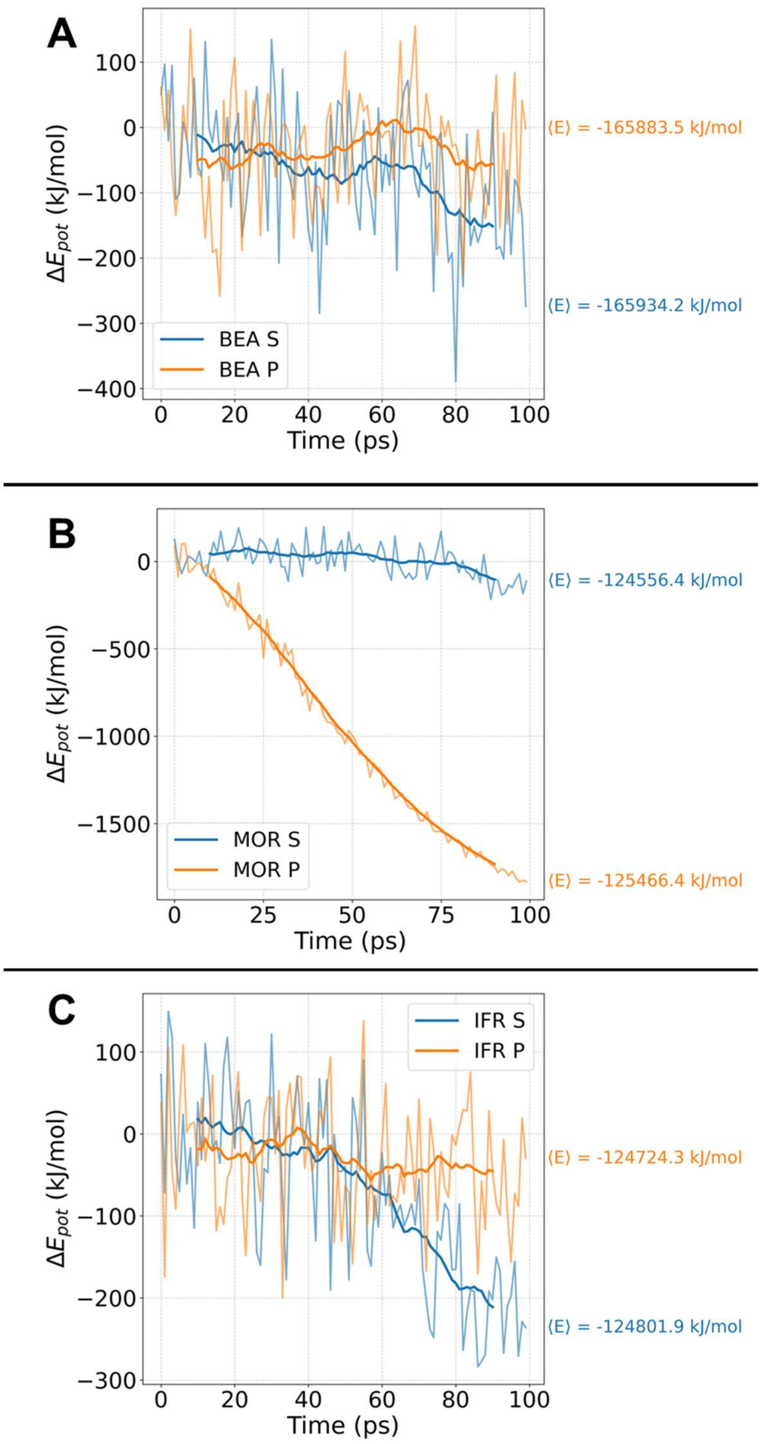

Figure S 5: Total bias-corrected potential energies of the umbrella windows (classical FF) at S (blue) and P (orange) in BEA (**A**), MOR (**B**) and IFR (**C**) during the production run, shifted to the average of the first 10 ps. For better visibility the running average over 20 ps is shown and for completeness the total average is shown on the right of the plot.

For MOR (Figure S 5 B) the bias-corrected potential energy at **S** oscillates around its initial value, indicating a well-equilibrated simulation. Prior to the production run at **P** the CBZ molecule was moved through the narrow part of the 12 MR channel. The energy during this production run is not stable but steadily decreases by about 20 kJ/mol per ps, which is a too

large and too slow change to be attributed to the CBZ molecule rearranging. In the initial state, the zeolite framework is apparently trapped in a metastable configuration and the significant deformation of the diffusion event allowed the framework to anneal into a new, more stable state, thus lowering the total potential energy. This can be further supported by evaluating the Si-O bond lengths in the trajectories of the production runs of the **S** and **P** configurations (Figure S 6). The average structures of the first and last 2.5 ps of the trajectories were analyzed for each configuration to make the effect of the framework relaxation more apparent.

The analysis of the trajectory (production run) at the initial adsorption site (configuration **S**) shows that the Si-O bond length distribution is broad and changes only minimally over the 100 ps production run. This indicates a kinetically trapped, strained, meta-stable zeolite structure that is persistent as long as no major structural perturbation occurs. The histogram for configuration **P** shows a similarly broad distribution in the beginning of the production run (red) but a significant relaxation of the framework towards less strained Si-O bonds over the course of the 100 ps production run. This is direct evidence that the local distortions of the framework, induced by the forced diffusion event, led to the annealing into a more stable, relaxed zeolite structure. From this we can infer that the initial configuration of the MOR framework obtained with the classical FF description is not the global minimum, demonstrating the vulnerability of this level of theory when it comes to complex, interconnected systems.

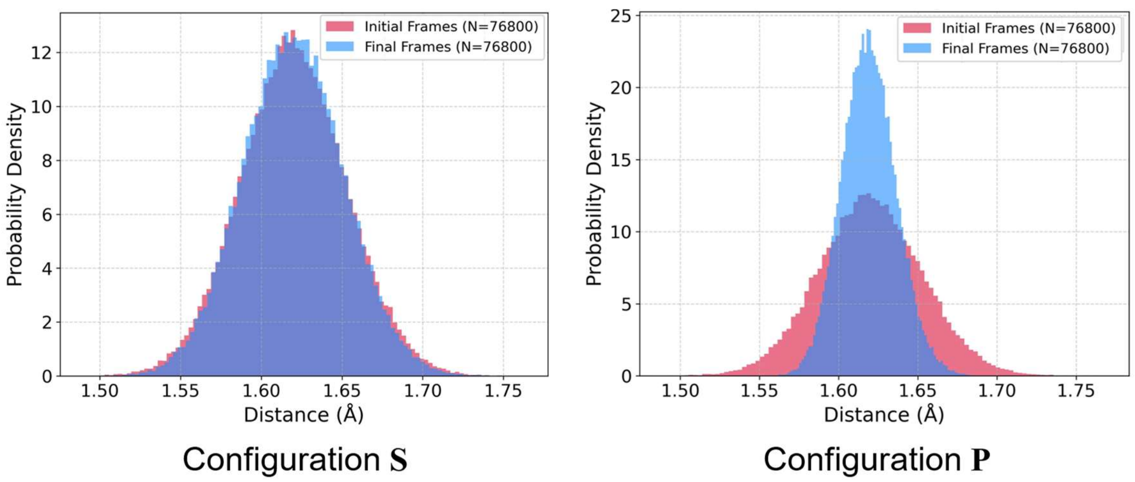

Figure S 6: Histogram of Si-O bond lengths during the production runs of the CBZ/MOR system in configuration **S** (left) and **P** (right). The analyzed structures are the average of the first 2.5 ps (red) and last 2.5 ps (blue).

The framework relaxation seems to create a local environment that is less favorable for the guest, resulting in a higher free energy for the guest at that specific point, possibly due to local rearrangements during the relaxation that disturb the CBZ molecule. This shows an interesting decoupling of the host and guest energetics, where the relaxation of the host does not necessarily correspond to an improved binding site for the guest, a nuance that would be impossible to uncover without a detailed analysis of both the total energy and the free energy

surface. Since the free energy difference along the diffusion coordinate is merely 20 kJ/mol, it is apparent that the framework relaxation, lowering the total potential energy by more than 1500 kJ/mol only has a minor effect on the adsorption/diffusion behavior.

In contrast to the slow relaxation of the whole zeolite framework observed in MOR, the IFR framework exhibits a different form of hysteresis. Figure S 5 C shows the time evolution of the bias-corrected total potential energy in the umbrella windows corresponding to the initial (**S**) and crystallographically equivalent (**P**) minimum. On average the potential energy of the **P** configuration is about 80 kJ/mol higher, which can likely be attributed to a local, slowly dissipating, high-energy strain on the IFR framework, induced by the forced diffusion jump.

Thus, we infer that to obtain a symmetric FES in the cases of the BEA and IFR frameworks, significantly longer simulation times would be required. The asymmetry is a result of kinetic trapping, following a diffusion event, the system becomes trapped in a strained, higher-energy metastable orientation. Because the rotational and framework relaxation times exceed the simulation window, the system is non-ergodic on this timescale and fails to converge to the global Boltzmann distribution at the crystallographically equivalent adsorption site. This might be due to the highly strained nature of these high-energy configurations in which the chosen classical FF parameters do not seem to perform well. In the MOR framework we argue that in addition to this general issue, the framework relaxation (see Figure S 6) further adds to the non-ergodicity.

Beyond the explanation of the asymmetric FES, the persistence of local distortions, possibly triggering rearrangements of the whole framework and different adsorption configurations at crystallographically equivalent sections of the pore have direct implications for the real-world diffusion kinetics. While a general rearrangement of the framework is likely an artifact of the classical forcefield description, local distortions or higher-energy adsorption configurations that follow a diffusion event can promote further diffusion, since the system is in a higher-energy state, effectively decreasing the energy between the diffusion barrier and the adsorption site. A simple TST calculation might use the barrier height relative to the global minimum, these results suggest that after an initial diffusion jump a small, yet significant population of higher-energy states can be occupied. The macroscopic diffusion coefficient, being an ensemble average over all possible kinetic pathways, is therefore likely to be higher than predicted by a model that only considers hopping between the lowest-energy ground states. Assessing the rotational barriers for the rotation of the whole molecule between adsorption configurations in the BEA framework as well as investigating the longevity and spatial propagation of local framework distortions can complete this understanding even more. Based on this data, kinetic Monte Carlo methods could be employed to move beyond TST for the prediction of diffusion coefficients.

S5. Comparison of classical FF and MACE free energy surfaces for the diffusion of CBZ in CFI, BEA, MOR and IFR

Figure S 7 shows the FES of the CBZ jump diffusion through CFI (A), BEA (B), MOR (C) and IFR (D). The classical FF and MACE level of theory are compared, as well as the effect of different production durations and equilibration strategies for the diffusion in MOR and IFR.

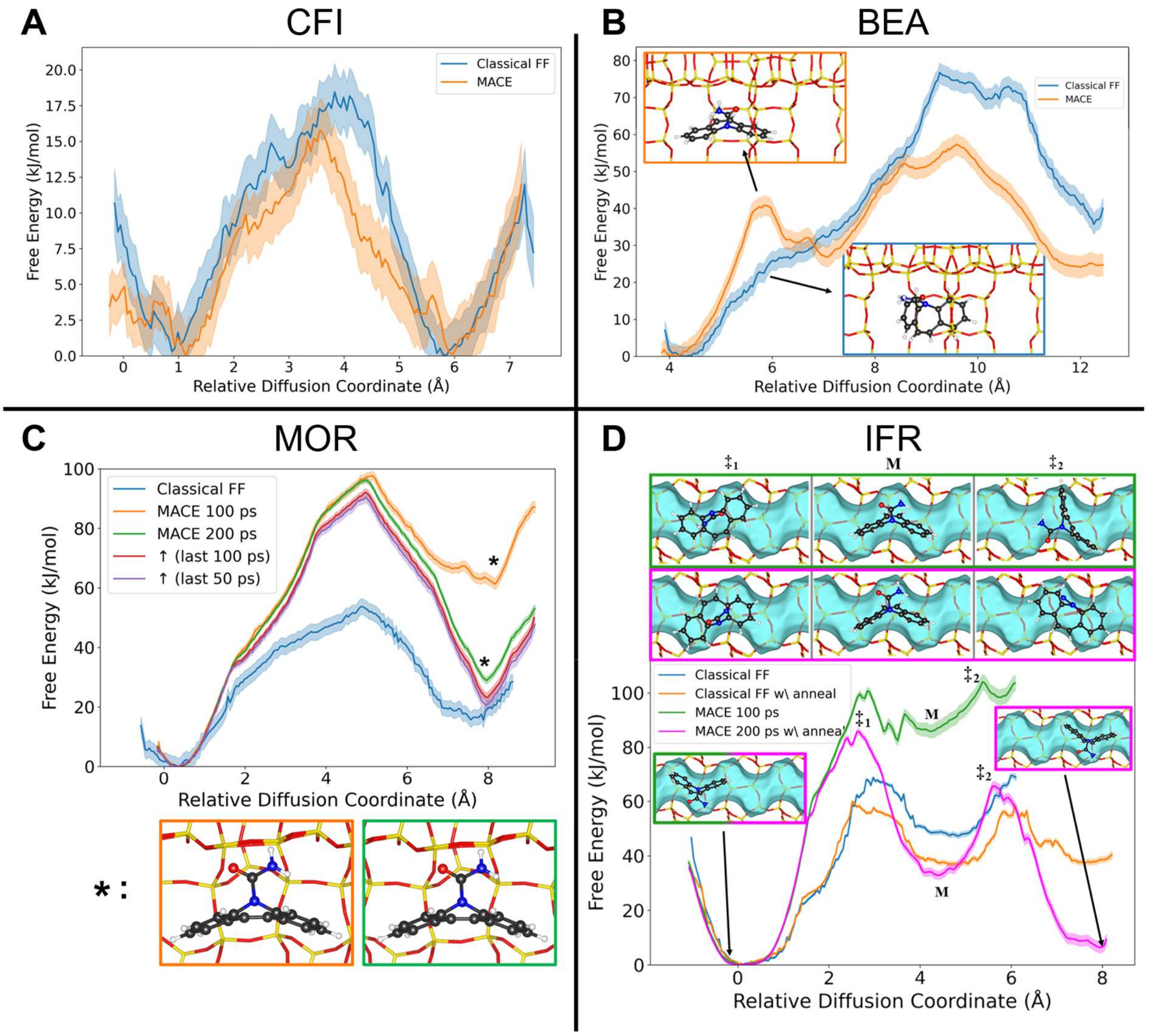

Figure S 7: **A**: FES of CBZ diffusing through CFI (blue: classical FF, orange: MACE) showing quantitative agreement. **B**: FES of CBZ diffusing through BEA (blue: classical FF, orange: MACE) with the ensemble-averaged configurations of CBZ at the first energy barrier of the MACE FES. **C**: FES of CBZ diffusing through MOR (blue: classical FF, orange/green/red/violet: MACE) with the ensemble-averaged configuration of CBZ on the orange and green FES at the second minimum. **D**: FES of CBZ diffusing through IFR (blue/orange: classical FF, green/magenta: MACE). The orange and magenta FES were obtained by employing an annealing procedure for equilibration. The averaged configurations are depicted at the respective position on the FES are shown, with the outline of the electron density of the zeolite framework shown in blue to enhance the visibility of the sinusoidal pores.

The FES for the diffusion in the wide 14 MR channels of CFI from both methods are almost identical (see Figure S 7 A). The location of the minima and the maximum of the FES agree very well, the activation barrier differs by ~3 kJ/mol, which is well within the errors of each

method and the MBAR analysis of the FES. In these channels, CBZ experiences almost no deformation, the configurations sampled are thus very close to the equilibrium state, a regime where both the classical force field and the MACE potential perform reliably and agree quantitatively.

In contrast, in the more constraining 12 MR pores of MOR, IFR and BEA, significant deviations are observed. The MACE simulations yield substantially higher energy barriers for the diffusion through MOR and IFR (Figure S 7 C/D), whereas for BEA (Figure S 7 B), the MACE barrier is lower than the classical FF one. We attribute this seemingly contradictory trend to the well-known shortcoming of the 12-6 LJ potential being too steep in the repulsive r^{-12} regime (overestimation of classical FF prediction for BEA) in conjunction with a small but systematic underestimation of interaction energies obtained from the classical FF simulations, from which we infer a slightly too shallow LJ potential well (underestimation of classical FF prediction for MOR and IFR). Generally, in these high-energy configurations the classical FF description is expected to fail, since its validity is limited to close-to-equilibrium configurations.[8] In the following each system is discussed individually.

This effect is most apparent in the BEA framework, which has the smallest LDS (5.5 Å). Here, the transition state imposes extremely close contacts between the guest and host. In this highly compressed regime, the classical FF's steep r^{-12} term overestimates the steric repulsion, leading to an artificially high activation barrier. The MACE potential, trained to reproduce the "softer" Pauli repulsion from DFT, yields a lower activation barrier. Additionally, the MACE FES features a distinct secondary barrier at ~2 Å, corresponding to the desorption the amide group out of an intersecting channel (Figure S 7 B, ensemble-averaged configurations). The averaged structures show that the classical FF avoids this steric hindrance by allowing the CBZ molecule to undergo an approximately 90° rotation along the channel axis. This enhanced rotational freedom in the classical FF simulation can be tracked back to the validation of the FF parameters in our previous work.[9] The LJ potential well of the dispersion parameters is slightly too shallow, evident from the slight underestimation of the interaction energies compared to DFT results. This leads to an artificially low kinetic barrier for rotating the molecule out of a favorable orientation. We conclude that this same circumstance is also responsible for the systematically lower diffusion barriers predicted by the classical FF for the MOR and IFR frameworks as well, where the LDS of 5.7 Å and 5.9 Å, respectively, does not result in those extremely close atomic contacts that lead to an overestimation of the classical FF. Also the adsorption configuration at the crystallographically equivalent section of the pore on the classical FF and MACE level of theory both show CBZ in a rotated orientation with respect to the initial adsorption configuration, which again explains the energy difference between the first and second minimum.

Considering the diffusion through MOR, the production time of 100 ps was long enough for CBZ to physically reach the low-energy configuration in the second minimum (Figure S 7 C, ensemble-averaged configuration, orange frame). However, the system seems to be non-ergodic in the minimum after the diffusion jump, leading to a higher energy at the crystallographically equivalent section of the pore. This skews the resulting probability distribution, leading to an artificially high free energy for the second minimum. Extending the simulation to 200 ps improved this, as it allows the system more time to physically relax out of the highly strained configurations following the diffusion jump. This longer sampling of the phase space provides a more converged FES and direct evidence of the slow relaxation dynamics on the MACE potential energy surface (see Figure S 7 C, green FES). Further analysis of the 200 ps production runs, only considering the last 100 ps (Figure S 7 C, red FES) and last 50 ps (Figure S 7 C, violet FES) shows a further decrease in the energy of the second minimum, indicating that with increasing simulation time both minima should become energetically equivalent, approaching ergodicity. Importantly, the height of the activation barrier remains consistent between the 100 ps and 200 ps simulations, with a slight lowering by less than 5 kJ/mol for the FES of the last 100 ps/ 50 ps of the 200 ps production run, which is negligible compared to the total height of the activation barrier. This indicates that the transition state region is sufficiently sampled within the shorter timeframe, and the slow convergence is a phenomenon specific to the post-barrier relaxation into the minimum. An inspection of the time-evolution of the bias-corrected potential energy at both minima of the 100 ps FES (Figure S 8), shows a stable oscillation in contrast to the classical FF simulation of MOR, indicating that the optimization with the MACE potential reliably converges into the global minimum.

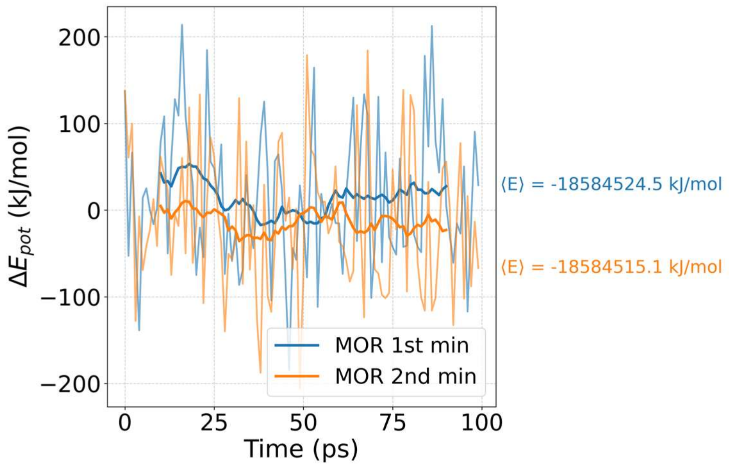

Figure S 8: Total bias-corrected potential energies of the umbrella windows of the CBZ-MOR system at the first minimum (blue) and at the second minimum (orange) during the production run of the 100 ps MACE simulation, shifted to the average of the first 10 ps. For better visibility the running average over 20 ps is shown and for completeness the total average is shown on the right of the plot.

The slow relaxation is even more pronounced for the diffusion of CBZ through the sinusoidal channels of IFR. The configurations after the first barrier crossing became trapped in high-energy, strained configurations (Figure S 7 D, green FES) when no additional equilibration strategy was employed. The time-evolution of the bias-corrected potential energy of the 100 ps run at the first and second minimum ($\mathbf{M}$) can be considered to demonstrate this. Figure S 9, top, directly shows the high-energy configurations at the start of the production run of the 100 ps simulation.

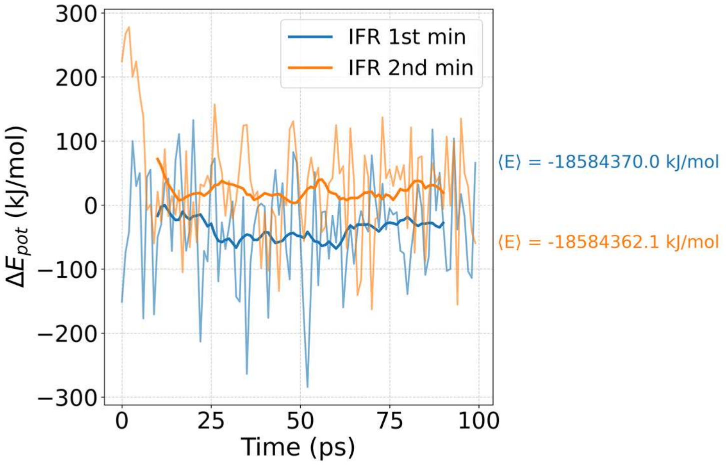

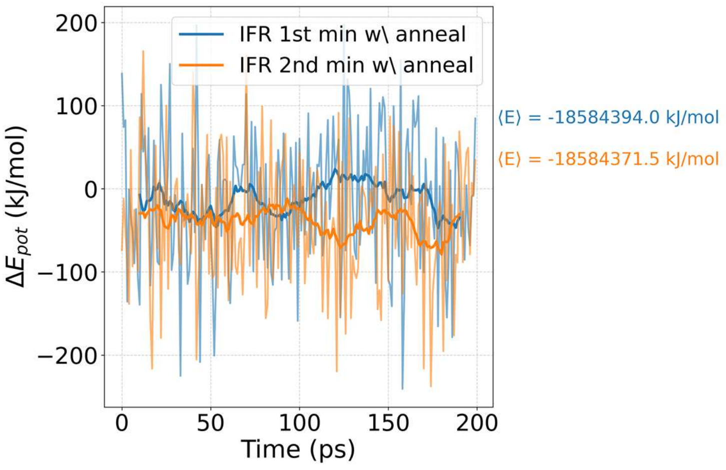

Figure S 9: Total bias-corrected potential energies in the CBZ/IFR system of the umbrella windows at the first minimum (blue) and at $\mathbf{M}$ (orange) during the production run of the 100 ps MACE simulation (Top) and the 200 ps MACE simulation, equilibrated with the annealing strategy (Bottom), shifted to the average of the potential energy between 10 and 20 ps. For better visibility the running average over 20 ps is shown and for completeness the total average is shown on the right of the plot.

The kinetic trapping is also apparent from the large autocorrelation times in the umbrella windows after the transition states (see ESI, IFR_ogre_MACE_100ps.dat) of up to 5 ps, giving only 20 independent configurations, leading to an insufficient representation within the umbrella window. To overcome this, an adapted equilibration protocol involving a

high-temperature annealing step was employed. This strategy successfully bypassed the slow rotational dynamics, allowing the system to sample the likely true, low-energy path. At the bottom of Figure S 9 the potential energy evolution of the respective umbrella windows on the magenta FES (Figure S 7 D), equilibrated with the annealing strategy, is shown. Here no pronounced high-energy states after the diffusion jump are visible.

The transition state of the green and the magenta FES are qualitatively similar, the average configurations (Figure S 7 D, $\ddagger_1$) look largely identical. Even though the average configurations at the second minimum (Figure S 7 D, **M**) look similar for the 100 ps run and the 200 ps + annealing run, they differ upon closer inspection, which is documented in Table S 2. From the larger angle and the systematically shorter bonds of configuration **M**/100 ps, it is apparent that the molecule experiences a large amount of strain, while it exists almost in its unconstrained equilibrium in configuration **M** of the 200 ps + annealing run, directly demonstrating the kinetic trapping without the annealing equilibration.

The configurations at the second transition state (Figure S 7 D, $\ddagger_2$) differ significantly, with the configuration $\ddagger_2$/100 ps being trapped “sideways” which prohibits further diffusion in contrast to $\ddagger_2$/200 ps + annealing which is largely symmetrically equivalent to $\ddagger_1$.

Table S 2: Analysis of the average structure of CBZ in the second minimum of the orange (100 ps) and red (200 ps + annealing) FES featuring the average bond lengths of C-H, C-C and C-N bonds, the C-O bond length, the C-N-C angle and the geometric axis of the CBZ molecule.

	CBZ in IFR Configuration **M**		
	MACE 100 ps	MACE 200 + anneal	CBZ unconstrained
Angle C-N-C (°)	123.13	116.27	116.78
C-H_{ave} (Å)	0.992	1.077	1.078
C-C_{ave} (Å)	1.309	1.393	1.395
C-N_{ave} (Å)	1.31	1.403	1.407
C-O (Å)	1.132	1.216	1.215
Axis 1 (Å)	9.239	9.216	9.418
Axis 2 (Å)	4.593	5.303	5.327
Axis 3 (Å)	4.748	5.161	5.114

To ensure the validity of the annealing strategy, classical FF simulations employing the same equilibration procedure were employed, the associated FES is shown in Figure S 7 D, orange FES. Both classical FF FES agree qualitatively and largely quantitatively with a consistent difference of around 10 kJ/mol after overcoming the first barrier. Compared to the total height of the barrier this is a minor deviation. However, we need to confirm that the observed ~10/20 kJ/mol (FF/MACE) lowering of the transition state (and the subsequent free energy

profile) is a consequence of improved sampling and not a thermodynamic artifact, which will be discussed subsequently.

The temperature dependence of the activation free energy is governed by the activation entropy ($\Delta G^{\ddagger}=\Delta H^{\ddagger}-T\Delta S^{\ddagger}$.). For diffusion in a confined environment, $\Delta S^{\ddagger}$ is expected to be negative, as the transition state is generally more ordered than the already constrained ground state. To establish a reasonable upper bound for the magnitude of this effect, we can consider literature values for related systems. Gounder *et al.* reported that the activation entropy for propane cracking becomes more negative by -26 J/(mol·K) when the reaction occurs in an 8 MR channel (2.3 Å LDS) compared to a 12 MR channel (5.7 Å LDS) in the MOR framework[10]. While this value represents a difference between two environments, it provides an illustrative benchmark for the magnitude of entropic penalties arising from extreme confinement.

In contrast, the diffusion of CBZ through the IFR framework involves a much more subtle change in pore diameter of only 0.6 Å (LIS_{IFR} - LDS_{IFR}). It is therefore unlikely that the absolute activation entropy for this less severe constriction would exceed the magnitude observed for the extreme case in the reference. Using -26 J/(mol·K) as a very generous upper bound for $\Delta S^{\ddagger}$, the expected thermodynamic change in the free energy barrier over the 300 K temperature difference would amount to only 7.8 kJ/mol. The larger difference of ~20 kJ/mol observed between the barrier of the MACE simulations with and without annealing therefore cannot be a simple thermal artifact. This strongly indicates that the annealing protocol is not introducing an error, but is instead correcting for a severe sampling deficiency by allowing the system to escape high-energy metastable states. Thus, we conclude that the FES obtained from the annealing equilibration strategy yield meaningful results for the diffusion under ambient conditions.

The evolution of the bias-corrected potential energy of the system is shown in Figure S 10 and shows a similar framework relaxation as the classical FF MOR system. Here the framework rearrangement was apparently triggered by the annealing step. This underlines once more the limitations of classical potentials to reliably describe complex interconnected structures like zeolite frameworks. From the analogue plot of the potential energy of the MACE simulations with the annealing step (Figure S 9) it is apparent that the MACE description yields a reliable description of the system with a stable total energy.

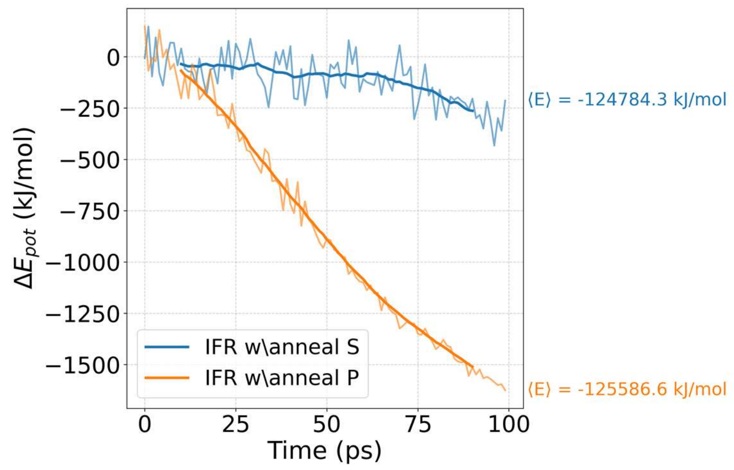

Figure S 10: Total bias-corrected potential energies of the umbrella windows of the CBZ-IFR system simulated with the annealing strategy on the classical FF level at the first minimum (blue) and at the second minimum (orange) during the production run, shifted to the average of the first 10 ps. For better visibility the running average over 20 ps is shown and for completeness the total average is shown on the right of the plot.

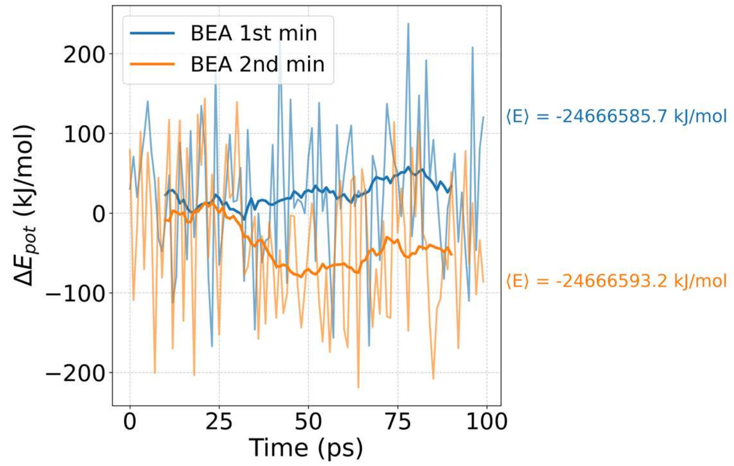

Figure S 11: Total bias-corrected potential energies of the umbrella windows of the CBZ-BEA system at the first minimum (blue) and at the second minimum (orange) during the production run of the 100 ps MACE simulation, shifted to the average of the first 10 ps. For better visibility the running average over 20 ps is shown and for completeness the total average is shown on the right of the plot.

As a measure for the flexibility and distortions of the zeolite frameworks and the internal geometry of the CBZ molecule during the diffusion process the root-mean-squared deviations (RMSD) of the atomic positions with respect to the center of mass of the framework, respectively the molecule, are shown in Table S 3. From the RMSDs the increased flexibility of the zeolite frameworks and the CBZ molecule in the MACE description is directly apparent.

Table S 3: Root-mean-squared deviation (RMSD) of the atomic coordinates (for the zeolites at the minima and the maxima of the FES and for CBZ along the whole FES) from the classical FF and MACE trajectories.

		Zeolite			
		CFI	BEA	MOR	IFR
Classical FF	$RMSD_{CBZ}$ (Å)	0.55	0.83	0.94	0.65
	$RMSD_{ZEO}$ (Å)	0.14	0.19	0.12	0.21
MACE	$RMSD_{CBZ}$ (Å)	1.26	2.80	1.64	2.02
	$RMSD_{ZEO}$ (Å)	0.46	0.45	0.35	0.24

S6. Estimating the transmission coefficient for CBZ in MOR and CFI

In the results section "Deducing diffusion properties from MACE activation free energies" a transmission coefficient of κ=1 is assumed. Here we connect Einstein diffusion with Kramer's reaction-rate theory to estimate κ from the friction between the zeolite and the molecule (γ) and the imaginary frequency of the transition state (ω_{TS}).[11,12] According to Kramer the transmission coefficient can be written as

$$\kappa = \frac{1}{\omega_{TS}}\left(-\frac{\gamma}{2}+\sqrt{\frac{\gamma^2}{4}+\omega_{TS}^2}\right). \qquad \text{(SI-Eq. 6)}$$

The imaginary frequency of the transition state (ω_{TS}) can be obtained from the curvature (*V''*) of the FES at the transition state and the mass of the CBZ molecule (*m*) as

$$\omega_{TS} = \sqrt{\frac{V''}{m}}. \qquad \text{(SI-Eq. 7)}$$

This was fitted including 10 datapoints in either direction around the transition state using a 2nd order polynomial (see Figure S 12 and Figure S 13). The friction between the zeolite and the CBZ molecule was calculated from the local self-diffusion coefficient in the umbrella window closest to the transition state. The local self-diffusion coefficient is related to the friction by

$$D_S = \frac{k_B T}{\gamma} = \frac{Var(\xi_{TS})}{\tau(\xi_{TS})} \qquad \text{(SI-Eq. 8)}$$

, and can be obtained from the variance and the autocorrelation time of the CV in the respective umbrella window ($Var(\xi_{TS})$).[13] We obtain an imaginary frequency of 3.89×10^{13} s^{-1} for the diffusion transition state of CBZ in CFI and a friction coefficient of 1.26×10^{13} s^{-1}. This results in a transmission coefficient of 0.85. For the MOR zeolite the imaginary frequency of the transition state is 8.45×10^{13} s^{-1} and the friction coefficient is 1.51×10^{13} s^{-1}, which gives a transmission coefficient of 0.91.

We can connect these values with our intuition for the interplay of CBZ with each pore. In the wider pores of CFI friction is lower than in the narrower pores of MOR. Due to the tighter squeeze that CBZ experiences at the transition state in MOR, the curvature and thus the imaginary frequency is higher than in CFI. In both cases however, the transmission coefficient is close to 1. Since the barrier differences between the zeolites influence the rate constant exponentially and the influence of the transmission coefficient is linear, we infer that the general trends we have reported still hold.

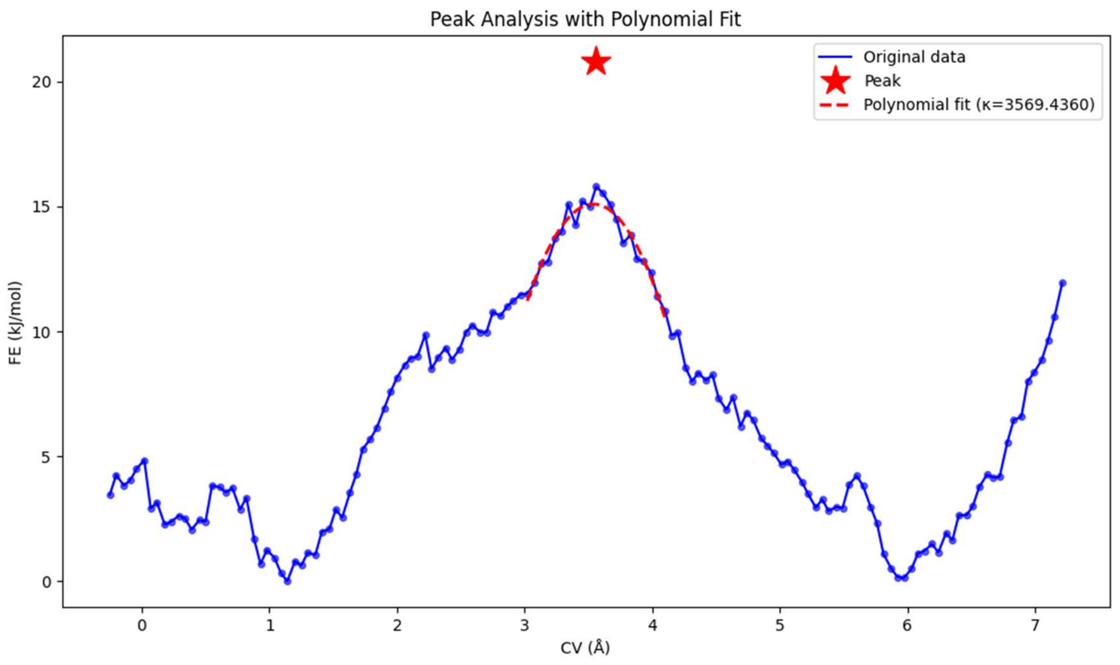

Figure S 12: FES of the diffusion of CBZ in the CFI zeolite calculated with MACE. A polynomial fit to obtain the curvature of the transition state is shown.

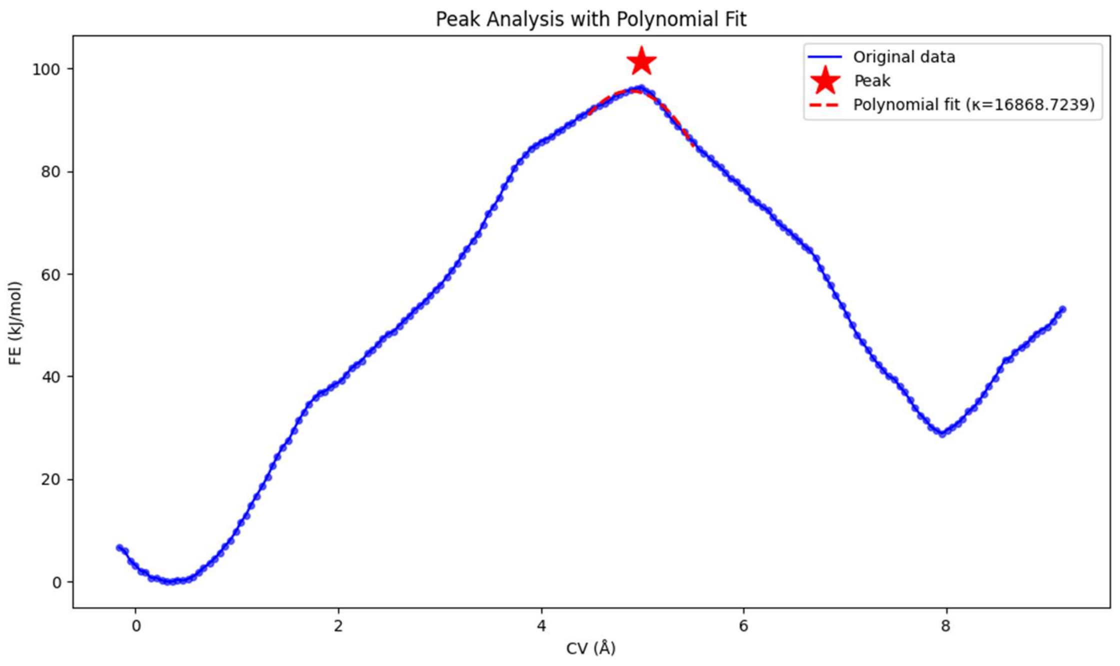

Figure S 13: FES of the diffusion of CBZ in the MOR zeolite calculated with MACE. A polynomial fit to obtain the curvature of the transition state is shown.

References

[1] T. D. Kühne, M. Iannuzzi, M. Del Ben, V. V. Rybkin, P. Seewald, F. Stein, T. Laino, R. Z. Khaliullin, O. Schütt, F. Schiffmann, D. Golze, J. Wilhelm, S. Chulkov, M. H. Bani-Hashemian, V. Weber, U. Borštnik, M. Taillefumier, A. S. Jakobovits, A. Lazzaro, H. Pabst, T. Müller, R. Schade, M. Guidon, S. Andermatt, N. Holmberg, G. K. Schenter, A. Hehn, A. Bussy, F. Belleflamme, G. Tabacchi, A. Glöß, M. Lass, I. Bethune, C. J. Mundy, C. Plessl, M. Watkins, J. VandeVondele, M. Krack, J. Hutter, "CP2K: An electronic structure and molecular dynamics software package - Quickstep: Efficient and accurate electronic structure calculations" *J. Chem. Phys.* **2020**, *152*, 194103.

[2] I. Hamada, "van der Waals density functional made accurate" *Phys. Rev. B* **2014**, *89*, 121103.

[3] M. Krack, "Pseudopotentials for H to Kr optimized for gradient-corrected exchange-correlation functionals" *Theor. Chem. Acc.* **2005**, *114*, 145–152.

[4] J. VandeVondele, J. Hutter, "Gaussian basis sets for accurate calculations on molecular systems in gas and condensed phases" *J. Chem. Phys.* **2007**, *127*, 114105.

[5] J. Li, J. Zhou, Y. Xiong, X. Chen, C. Chakrabarti in *2022 IEEE Workshop on Signal Processing Systems (SiPS)*, IEEE, **2022**, pp. 1–6.

[6] I. Batatia, P. Benner, Y. Chiang, A. M. Elena, D. P. Kovács, J. Riebesell, X. R. Advincula, M. Asta, M. Avaylon, W. J. Baldwin, F. Berger, N. Bernstein, A. Bhowmik, F. Bigi, S. M. Blau, V. Cărare, M. Ceriotti, S. Chong, J. P. Darby, S. De, F. Della Pia, V. L. Deringer, R. Elijošius, Z. El-Machachi, F. Falcioni, E. Fako, A. C. Ferrari, J. L. A. Gardner, M. J. Gawkowski, A. Genreith-Schriever, J. George, R. E. A. Goodall, J. Grandel, C. P. Grey, P. Grigorev, S. Han, W. Handley, H. H. Heenen, K. Hermansson, C. Holm, C. H. Ho, S. Hofmann, J. Jaafar, K. S. Jakob, H. Jung, V. Kapil, A. D. Kaplan, N. Karimitari, J. R. Kermode, P. Kourtis, N. Kroupa, J. Kullgren, M. C. Kuner, D. Kuryla, G. Liepuoniute, C. Lin, J. T. Margraf, I.-B. Magdău, A. Michaelides, J. H. Moore, A. A. Naik, S. P. Niblett, S. W. Norwood, N. O'Neill, C. Ortner, K. A. Persson, K. Reuter, A. S. Rosen, L. A. M. Rosset, L. L. Schaaf, C. Schran, B. X. Shi, E. Sivonxay, T. K. Stenczel, V. Svahn, C. Sutton, T. D. Swinburne, J. Tilly, C. van der Oord, S. Vargas, E. Varga-Umbrich, T. Vegge, M. Vondrák, Y. Wang, W. C. Witt, T. Wolf, F. Zills, G. Csányi, "A foundation model for atomistic materials chemistry" **2025**, DOI 2401.00096.

[7] E. Kasoar, P. Austin, H. Devereux, K. Harris, D. Mason, J. Wilkins, F. Zanca, A. Elena, **2025**, Zenodo preprint, DOI: https://doi.org/10.5281/zenodo.17064092.

[8] F. Jensen, *Introduction to Computational Chemistry*, Wiley Inc., **2006**.

[9] J. Brauer, M. Fischer, “Computational Screening of Hydrophobic Zeolites for the Removal of Emerging Organic Contaminants from Water” *ChemPhysChem* **2024**, *25*, e202400347.

[10] R. Gounder, E. Iglesia, “The Roles of Entropy and Enthalpy in Stabilizing Ion-Pairs at Transition States in Zeolite Acid Catalysis” *Acc. Chem. Res.* **2012**, *45*, 229–238.

[11] P. Hänggi, P. Talkner, M. Borkovec, “Reaction-rate theory: fifty years after Kramers” *Rev. Mod. Phys.* **1990**, *62*, 251–341.

[12] H. A. Kramers, “Brownian motion in a field of force and the diffusion model of chemical reactions” *Physica* **1940**, *7*, 284–304.

[13] G. Hummer, “Position-dependent diffusion coefficients and free energies from Bayesian analysis of equilibrium and replica molecular dynamics simulations” *New J. Phys.* **2005**, *7*, 34–34.
